# Post-translational buffering leads to convergent protein expression levels between primates

**DOI:** 10.1186/s13059-018-1451-z

**Published:** 2018-06-27

**Authors:** Sidney H. Wang, Chiaowen Joyce Hsiao, Zia Khan, Jonathan K. Pritchard

**Affiliations:** 1 Center for Human Genetics, The Brown foundation Institute of Molecular Medicine, The University of Texas Health Science Center at Houston, Houston, TX USA; 2 Department of Human Genetics, University of Chicago, Chicago, IL USA; 3 Genentech, 1 DNA Way, South San Francisco, CA USA; 4 Department of Genetics, Stanford University, Stanford, CA USA; 5 Department of Biology, Stanford University, Stanford, CA USA; 6 Howard Hughes Medical Institute, Stanford University, Stanford, CA USA

**Keywords:** Buffering, Translation, Ribosome profiling, Primate evolution, Gene regulation

## Abstract

**Background:**

Differences in gene regulation between human and closely related species influence phenotypes that are distinctly human. While gene regulation is a multi-step process, the majority of research concerning divergence in gene regulation among primates has focused on transcription.

**Results:**

To gain a comprehensive view of gene regulation, we surveyed genome-wide ribosome occupancy, which reflects levels of protein translation, in lymphoblastoid cell lines derived from human, chimpanzee, and rhesus macaque. We further integrated messenger RNA and protein level measurements collected from matching cell lines. We find that, in addition to transcriptional regulation, the major factor determining protein level divergence between human and closely related species is post-translational buffering. Inter-species divergence in transcription is generally propagated to the level of protein translation. In contrast, gene expression divergence is often attenuated post-translationally, potentially mediated through post-translational modifications.

**Conclusions:**

Results from our analysis indicate that post-translational buffering is a conserved mechanism that led to relaxation of selective constraint on transcript levels in humans.

**Electronic supplementary material:**

The online version of this article (10.1186/s13059-018-1451-z) contains supplementary material, which is available to authorized users.

## Background

Almost half of a century ago, King and Wilson postulated that gene regulation differences are the major factor driving phenotypic divergence between human and chimpanzee [1]. Indeed, differences in gene regulation have been reported to be the major factors determining phenotypic differences between closely related species [2, 3]. Alterations of gene expression levels are more likely to survive natural selection than coding substitutions since a limited spatiotemporal change in gene expression is less likely to have deleterious pleotropic effects. Multiple examples, e.g. pelvic fin reduction in freshwater sticklebacks, demonstrate how changes in gene expression patterns could result in dramatic phenotypic divergence between closely related species [4]. Over the past decade, studies surveying genome-wide gene expression levels in primates have documented substantial variation in transcript levels between closely related species. [5–8]. Furthermore, population genomics studies looking for signatures of recent selection also highlighted key roles of regulatory variants in human adaptation [9, 10]. Despite promising progress, how variation at the transcript level impacts evolution of an organismal trait remains far from clear.

Protein expression levels are the biologically relevant quantities for coding genes. Yet most studies investigating divergence in gene expression among primates focused on comparing expression levels of messenger RNA (mRNA) [5, 6, 11, 12]. While the general efficacy of using mRNA level as a proxy for estimating protein levels is still an ongoing debate [13], there is no doubt that in some instances, translational and/or post-translational regulation of gene expression results in protein levels that are far deviated from the mRNA levels upstream [14]. Large-scale studies profiling the impact of genetic variation jointly on transcript and protein levels have begun to reveal clues on how protein expression is post-transcriptionally regulated [15–18]. In fact, it has been shown that protein expression levels are far more conserved across diverse taxa than mRNA levels [19]. How this conservation of protein level is achieved given the apparent larger divergence at the mRNA level is still unclear.

To estimate the divergence of mRNA and protein levels among primates, we previously collected RNA-sequencing and quantitative mass spectrometry data from a set of 15 primate (five humans, five chimpanzees, and five rhesus macaques) lymphoblastoid cell lines (LCLs). Consistent with an earlier observation across wider taxa [19], we found conserved protein expression levels between human and chimpanzee, despite extensive divergence in mRNA levels [20]. While a stronger evolutionary constraint at the protein expression level likely reflects the critical importance of protein stoichiometry [18], which is critical for forming the machinery that executes biological functions, it remains unclear how the divergence is buffered. More specifically, whether this buffering occurs translationally or post-translationally remains an open question.

Ribosome profiling is a technique that uses next generation sequencing to survey ribosome footprints in a massively parallel fashion [21]. It has been shown that ribosome occupancy levels estimated from counting the number of ribosome footprints provide a good approximation for the level of protein translation [22]. Several studies have successfully applied ribosome profiling to a wide range of organisms to better understand expression divergence across species and the impact of genetic variation within species [17, 22–28]. We recently applied this technique in a panel of HapMap cell lines to identify genetic variants affecting protein translation and to estimate relative contributions of translational and post-translational regulation to steady state protein levels [17]. We found that among the human cell lines, protein levels are usually less variable than mRNA levels. Interestingly, variation at ribosome occupancy level is mostly in concordance with mRNA rather than protein levels. Concordance between transcription and translation indicates that the attenuation of transcript level variation in humans is mainly mediated by a mechanism downstream of protein translation.

To further investigate gene expression divergence across primates, we performed ribosome profiling experiments in human, chimpanzee, and rhesus macaque cell lines. By integrating previously published ribosome profiling [17], RNA sequencing (RNA-seq) and quantitative mass spectrometry data [20], we compared the relative contributions of transcriptional, translational and post-translational regulation to gene expression divergence. This dataset allowed us to interrogate the relationships between different layers of gene regulation and their roles in primate evolution. Results from this joint analysis suggested that post-translational buffering plays a major role in maintaining conserved protein levels across primates. To our knowledge, the current study offers the first global view of the translational landscape across primates.

## Results

To comparatively estimate levels of protein translation in primates we used ribosome profiling to sequence ribosome protected fragments (RPF) of mRNA [21]. We collected ribosome profiling data from LCLs of four humans, four chimpanzees, and four rhesus macaques (Additional file 1: Table S1) where data for estimating mRNA levels by RNA-seq and protein levels by SILAC were also available.

**Fig. 1:**
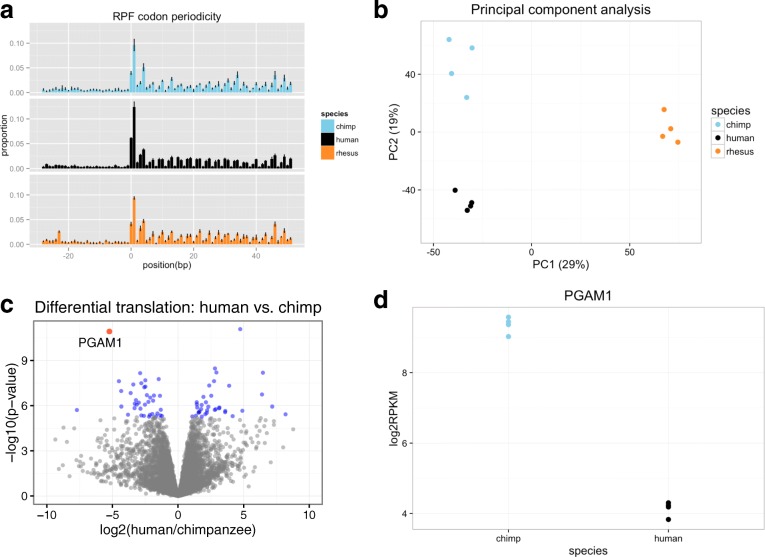
Inter-species comparison of protein translation among primates. **a** Subcodon periodicity pattern of ribosome footprints. Relative enrichment of RPF in an 80-bp window is aggregated along genomic positions surrounding conserved translation initiation sites (see “Methods” for details). *Bar height* represents mean ± standard error estimated from biological replicates for each species. **b** Major variation in level of protein translation reflects species differences. A *scatter plot* showing ribosome profiling data projected onto the first two PCs: each data point represents an individual sample. **c** Divergence in level of protein translation between human and chimpanzee. Each data point represents a gene: position along the *x-axis* indicates log_2_ ratio of ribosome occupancy level between human and chimpanzee, position along the *y-axis* indicates significance level, and the *color* of each data point indicates whether the gene is significantly diverged between species at a significance cut-off of FWER 0.05 (*blue*: significant, *gray*: not significant, *red*: the example significant gene shown in **d**). **d** Level of protein translation of an example gene. Ribosome occupancy level (log_2_ RPKM) of *PGAM1* is shown for each individual human and chimpanzee sample

After excluding sequencing reads that mapped to ribosomal RNA (rRNA) and other contaminating sources (see “Methods”), we obtained a median of ~ 12 million uniquely mapped ribosome profiling sequencing reads per sample (Additional file 1: Table S1 and Additional file 2: Figure 1 S1a). We performed several analyses to confirm that the quality of the data is consistently high across the three species (See “Methods” and Additional file 2: Figures 1 S1–S4). Briefly, we confirmed that > 95% of reads have a Phred quality score > 30 in all samples (Additional file 2: Figure 1 S1b), and that across samples, regardless of species, we observe a median footprint length of 29 nt (Additional file 2: Figure 1 S2) and a consistent codon periodicity pattern, as expected for ribosome profiling data (Fig. 1a). We also confirmed that technical variation (among different sequencing runs of the same sample) was significantly lower (*P* < 10^−15^, Wilcoxon rank sum) than biological variation (among different individuals from the same species; Additional file 2: Figure 1 S3). Finally, we explored the possibility that technical variables (such as sequencing depth and quality) could contribute to variation in our expression data. We determined that none of the technical variables we examined significantly contribute to variation in the normalized expression data (see “Methods” and Additional file 2: Figure 1 S4).

### Inter-species variation in levels of protein translation

To compare levels of translation across species, we focused our analysis on ribosome profiling sequencing reads that aligned to orthologous exons across the three species (used in [29]; Additional file 3). We combined data across technical replicates (i.e. pooling sequencing reads across all sequencing runs of the same sample) and normalized the sum of read counts across orthologous exons to estimate gene-specific levels of translation in each sample (see “Methods”). To examine global patterns in the data, we performed principal component analysis (PCA) on the gene-specific estimates of translation levels (Fig. 1b) and considered hierarchical clustering of pairwise Spearman’s rank correlation coefficients across samples (Additional file 2: Figure 1 S5). As expected, we found that the major source of variation between samples can be attributed to species, and that estimates of translation levels are more highly correlated between human and chimpanzee than between human (or chimpanzee) and the more distantly related rhesus macaque (as expected from the phylogeny of these three species; Fig. 1b, Additional file 2: Figure 1 S5).

To identify specific genes whose translation levels differ between species, we tested for each gene the association between species label and translation level using a linear model (see “Methods”). Differential translation, in this context, reflects the combined effects of differences in mRNA levels and in translation efficiency, because at this step, we have not yet accounted for inter-species differences in transcript levels. We first considered normalized ribosome profiling data from 9364 genes that were reliably quantified in all three species (Additional file 4, see “Methods” for criteria). At a family-wise error rate (FWER) of 5%, we classified 73 genes as differentially translated between humans and chimpanzees (Fig. 1c, d and Additional file 5). At the same FWER, we found 247 genes that are differentially translated between human and rhesus macaque. We also found 262 genes to be differentially translated between chimpanzee and rhesus macaque (Additional file 2: Figure 1 S6, Additional file 5). The number of differentially translated genes identified here again reflects the known phylogenetic distances between these species. Similar results were observed when either gender effects were removed as a potential confounding factor (Additional file 2: Figure 1 S7, see “Methods” for details) or inter-species footprint mappability differences were accounted for (Additional file 2: Figure 1 S8, see “Methods” for details). We next considered genes that are only quantifiable in a subset of species (see “Methods” for criteria). These genes are presumably differentially translated between species, since the level of translation is detectable only in one or two out of the three species considered. We identified 1287 genes that fall in this category (Additional file 6). Of these 1287 genes, we found 101 genes that are translated at a level only detectable in human, 52 genes that are only detectable in chimpanzee, and 81 genes that are only detectable in rhesus macaque (Additional file 6).

### Estimating divergence in translation efficiency

To evaluate the contribution of translational regulation to overall inter-species regulatory divergence, we analyzed the comparative ribosome profiling data in conjunction with corresponding RNA-seq and quantitative mass spectrometry data (from [20]). Since the cell lines used for ribosome profiling experiments described above does not match exactly the cell lines Khan et al. used to estimate transcript level and protein level [20], we collected ribosome profiling data from additional cell lines to match with protein and RNA datasets (see Additional file 1: Tables S1 and S7 for a full list of cell lines used in this study). We adjusted for potential artifacts that could be introduced by differences in processing batch using ComBat [30] (see “Methods”). Our goal was to estimate and compare the contribution of inter-species differences in translation to the observed attenuation of inter-species divergence at protein levels [20]. As a first step, we considered inter-species differences in translation efficiency, namely differences in translation level between species, which cannot be explained by corresponding inter-species differences in transcript levels. We tested for inter-species differences at the translation level that were significantly larger or smaller than inter-species differences at the transcript level (see “Methods” for details).

**Fig. 2:**
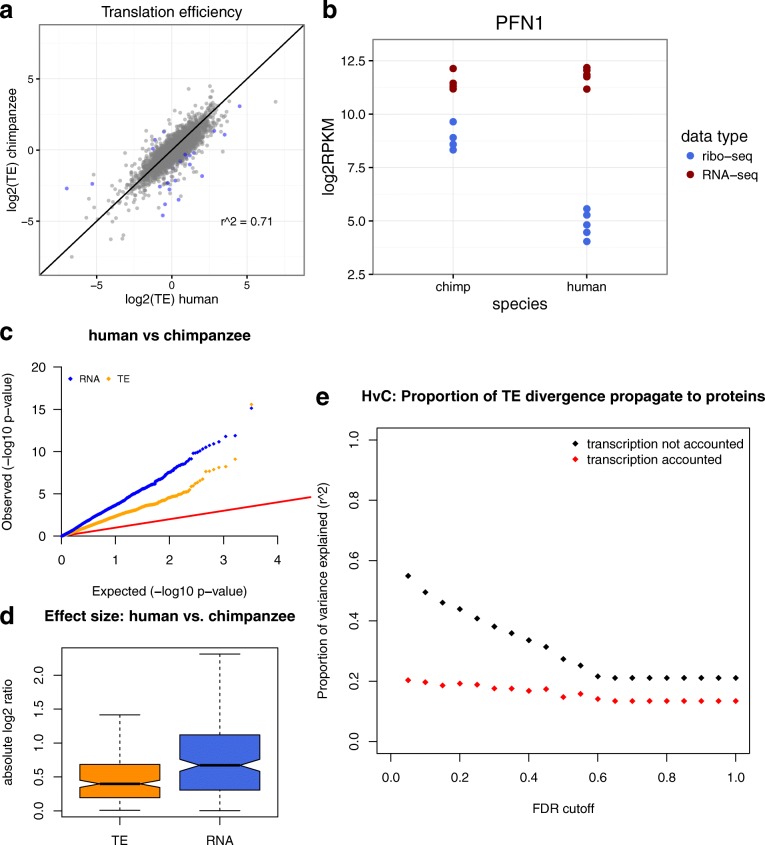
Transcriptional regulation contributes significantly more to protein level divergence compared to translational regulation. **a** Inter-species divergence in translation efficiency. A *scatter plot* comparing translation efficiency (TE) between human and chimpanzee. Each data point represents a gene, position along each axis indicates log_2_ translation efficiency of each species, and the *color* of each data point indicates whether the gene is significantly diverged between species in translation efficiency at a significance cut-off of FWER 0.05 (*blue*: significant, *gray*: not significant). **b** An example gene, Profilin 1 (*PFN1*), which shows human–chimpanzee divergence in translation efficiency. Level of protein translation (*blue*) and RNA transcription (*red*) are shown in log_2_RPKM. Each data point represents an individual human or chimpanzee sample. **c** Divergence between human and chimpanzee at the transcript level occurs more frequently than that of translation efficiency. Quantile-quantile plot of –log_10_(*p* values) derived from testing for divergence between human and chimpanzee for each trait of interest (RNA: transcript level, TE: translation efficiency). For each molecular trait, observed *p* value (*y-axis*) is plotted against the null expectation (i.e. uniform distribution of *p* values) (*x-axis*). The *red line* marks the expected results from a scenario where no divergence is observed. **d** Divergence between human and chimpanzee at the transcript level is greater than that of translation efficiency. *Boxplots* comparing effect size (absolute log_2_ ratio) of human-chimpanzee divergence (RNA: transcript level, TE: translation efficiency). Only genes that are diverged in protein levels were considered in this analysis. **e** Between human and chimpanzee, inter-species divergence in translation efficiency contributes little to inter-species divergence in protein level. Proportion of inter-species divergence propagated from translation level to the protein level (*y-axis*) was estimated using coefficient of determination (r^2^) between translation level divergence and protein level divergence. Each r^2^ was calculated for a subset of genes each defined by an FDR cut-off (*x-axis*) for divergence in protein level. These coefficients (r^2^) were calculated either with (*red*) or without (*black*) accounting for the effects from the transcript level

To facilitate a joint analysis combining data from all three molecular phenotypes, we focused on a set of 3286 genes for which we were able to obtain measurements across all three datatypes (mRNA, protein, and ribosome profiling) from at least three individuals for each species (Additional file 7). Between human and chimpanzee, we identified (at 5% FWER) a small number of 23 genes that are divergent in translation efficiency (Fig. 2a, b). Similarly, at 5% FWER we found 35 and 69 genes that are divergent in translation efficiency between rhesus–chimpanzee comparison and rhesus–human comparison, respectively (Additional file 2: Figure 2 S1). Thus, only a relatively small proportion (0.7–2.1%) of tested genes shows significant (5% FWER) divergence in translation efficiency. The scarcity of significant divergence in translation efficiency is in contrast to the level of divergence found in transcription (Fig. 2c, Additional file 2: Figure 2 S2). Furthermore, this contrast is not simply reflecting higher technical noise in estimating translation efficiency. For example, when considering genes that are diverged at protein level, we found that the effect size of inter-species divergence in translation efficiency is significantly smaller than that of transcription (human vs chimpanzee *P* < 10^−7^, rhesus vs chimpanzee *P* < 10^−15^, human vs rhesus *P* < 10^−15^, Wilcoxon rank sum) (Fig. 2d, Additional file 2: Figure 2 S3). Taken together, these results indicated that in contrast to divergence in gene regulation at the transcript level, divergence at the translational level has significantly less impact on inter-species divergence at the protein level.

To further evaluate the downstream impact of inter-species divergence at the translational level, we asked how often effects from divergence in translation efficiency propagate downstream to the protein level. After accounting for pre-existing divergence at the transcript level by regressing out transcript level divergence, we used the coefficient of determination (i.e. r^2^ between inter-species divergence in protein level and inter-species divergence in protein translation) to estimate the proportion of variance in protein level (quantitative mass spectrometry) that can be explained by translation (ribosome profiling). When all 3286 genes were considered (i.e. all genes that were sufficiently quantified across all three data types), we found relatively weak associations (r^2^ of 0.13 between human and chimpanzee, 0.01 between rhesus macaque and chimpanzee, and 0.07 between human and rhesus macaque). Because technical variation could dilute correlations, especially by introducing random noise in genes that are not diverged, we further evaluated this association by focusing on genes that are diverged between species in protein levels. After transcript level divergence was accounted for, weak associations between protein level and level of protein translation were consistently observed across different significance cut-offs (i.e. significance cut-offs for protein level divergence) (Fig. 2e, Additional file 2: Figure 2 S4). Taken together, these results indicate that for genes that are truly divergent at the protein level, only a small proportion (< 20%) of these effects are contributed by divergence in translation efficiency. Instead, the majority of these effects are contributed by divergence at the transcript level (Fig. 2e, Additional file 2: Figure 2 S4). These results confirmed the above observation that inter-species divergence in translation efficiency has only minor impact on divergence at the protein level.

### Attenuation of regulatory divergence

We previously reported that inter-primate divergence in mRNA levels is often attenuated at the protein level [20]. We observed a similar pattern with respect to variation in mRNA and protein expression levels within a human population [17]. Since intra-species genetic variation is relatively recent, we reason that compensatory mutations are less likely to mediate the observed attenuation. Instead, we proposed that downstream buffering mechanisms could modulate gene expression divergence introduced at the mRNA level. However, it remains unclear how such buffering is achieved. One intuitive hypothesis is that regulatory divergence at the transcript level is attenuated, or buffered, at translation. Alternatively, these effects could be buffered post-translationally at the protein level.

**Fig. 3:**
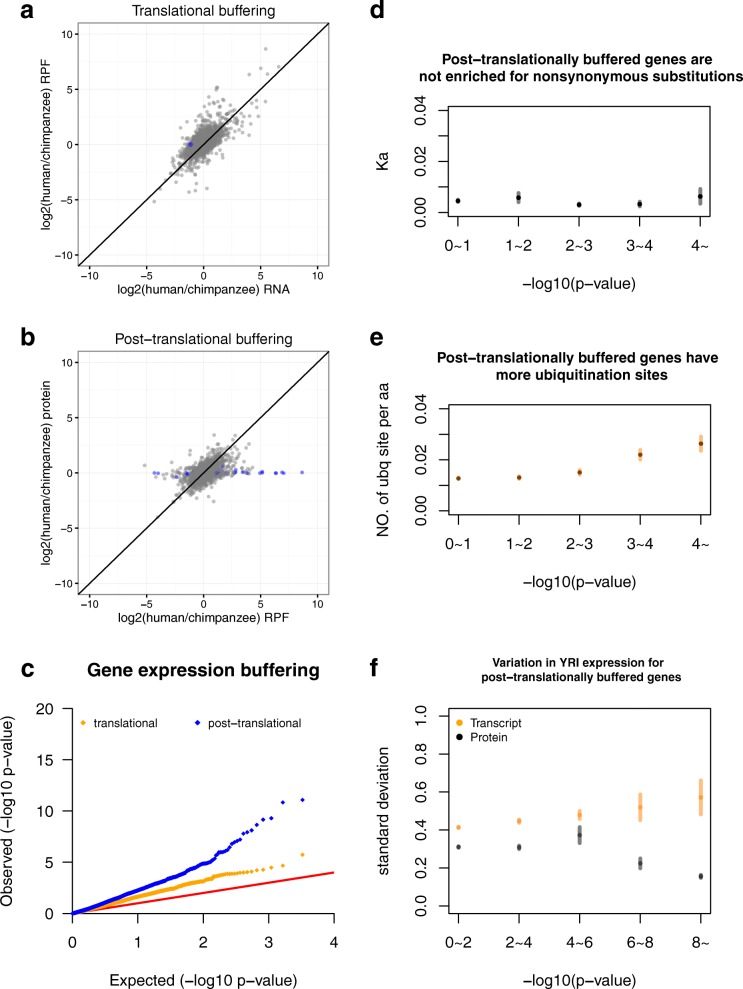
Gene expression buffering mainly occurs post-translationally and buffered genes are enriched for post-translational modifications. **a**, **b** *Scatter plots* of inter-species divergence comparing between different molecular traits (RNA: transcript level, RPF: level of translation, protein: protein level). Each data point represents a gene and the position along each axis indicates the log_2_ ratio between human and chimpanzee for each molecular trait. The *color* of each data point indicates whether the inter-species divergence for each gene is significantly buffered at the downstream molecular trait at a significance cut-off of FWER 0.05 (*blue*: significant, *gray*: not significant). **c** Post-translational buffering of human–chimpanzee divergence occurs much more frequently than translational buffering. *Quantile-quantile plot* of –log_10_(*p* values) derived from testing for buffering of human–chimpanzee divergence (*orange*: translational buffering, *blue*: post-translational buffering). Observed *p* values (*y-axis*) were plotted against the null expectation (i.e. uniform distribution of *p* values) (*x-axis*). The *red line* marks the expected results from a scenario where no buffering was observed. **d**, **e**, **f** Post-translationally buffered genes are enriched for post-translational modifications and a higher within-species transcript level variation. Individual genes were grouped into bins according to their significance level of human–chimpanzee post-translational buffering (*x-axis*). Position of each data point along the *y-axis* indicated mean ± standard error. **d** Post-translationally buffered genes are not significantly enriched for amino acid substitutions. Ka (proportion of nonsynonymous substitutions out of all possible non-synonymous sites) calculated between human and chimpanzee was plotted against significance level of human–chimpanzee post-translational buffering. **e** Post-translationally buffered genes have more ubiquitination sites. Number of reported ubiquitination sites in human was plotted against significance level of human–chimpanzee post-translational buffering. **f** Potential impact of post-translational buffering on relaxation of transcriptional regulation. Post-translational buffering of inter-species divergence is more likely to occur to genes that have a higher within-species (human) variation at the transcript level. Standard deviation across YRI individuals (reflects level of variation in the population) of transcript level (*orange*) or that of protein level (*black*) was plotted against significance level of human–chimpanzee post-translational buffering

To estimate the relative contributions of these two potential mechanisms, we devised a regression approach to identify divergence in gene expression that is lost in the downstream molecular phenotype. While buffering could alternatively be defined as a decrease in effect size at the downstream level (as was done by Khan et al. [20]), we chose to take the regression approach in order to identify only genes that have no remaining divergence at the downstream level. This regression approach is more conservative and allowed us to focus our analysis on buffering events that are more likely to be biologically relevant. For translational buffering, we tested for genes that are diverged at the transcript level, but not at the level of translation. Similarly, for post-translational buffering, we tested for genes that are diverged in level of translation, but not at the protein level. For each pair of molecular phenotypes, we first regressed out downstream effects from the upstream phenotype and then tested for divergence between species on residual effects (see “Methods” for details). Between human and chimpanzee, at 5% FWER, we found only one gene that is under translational buffering (Fig. 3a), while in contrast, 35 genes were found buffered post-translationally (Fig. 3b). Similar contrasts were observed for comparisons between human and rhesus and between chimpanzee and rhesus (translational vs post-translational: 7 vs 57 for human–rhesus comparison and 9 vs 45 for chimpanzee–rhesus comparison) (Additional file 2: Figure 3 S1). Moreover, we found consistently higher proportion of post-translational buffering than translational buffering when inspecting the full spectrum of the *p* value distribution (Fig. 3c, Additional file 2: Figure 3 S2). These results clearly indicated that post-translational buffering occurs much more frequently then translational buffering. Post-translational buffering is therefore the major force that attenuates divergence between species at the transcript level.

Post-translational mechanisms are often regulated in *cis*, determined by the composition of the protein sequence itself. We thus hypothesized that post-translationally buffered proteins would possess distinct features in their amino acid sequence or chemical composition. To explore enrichment of different properties of protein composition in this group of genes, we first addressed two potential biases commonly presented in enrichment analysis, i.e. gene length and GC content [31, 32]. We found little to no correlation between each of these two features and the significance value of post-translational buffering across genes (r^2^ < 0.01 in all pairwise species comparisons, Additional file 2: Figure 3 S3). We therefore decided to proceed with enrichment analysis without adjusting for these two features (see “Methods”). When considering the level of overall sequence divergence, we found no enrichment in post-translationally buffered genes in their proportion of non-synonymous substitutions for each pairwise species comparison (Fig. 3d, Additional file 1: Table S2). Although buffered genes are not enriched for inter-species amino acid substitutions, we next considered if they are more often targets of post-translational modifications and could therefore be differentially regulated post-translationally. We found significant enrichments of reported ubiquitination sites (*P* < 10^−15^) (Fig. 3e) and acetylation sites (*P* < 10^−7^) in post-translationally buffered proteins (Additional file 1: Table S3). We also found marginally significant enrichments of phosphorylation sites (*P* < 0.05), methylation sites (*P* < 0.05), and sumoylation sites (*P* < 0.01) in these proteins (Additional file 1: Table S3). These results indicated that although buffered genes are not particularly diverged in their amino acid sequence, they are enriched for post-translational modification sites and could therefore be differentially regulated via differential post-translational modifications between species. In addition to post-translational modifications on proteins, subcellular localization has been shown to impact protein turnover rate [33]. We therefore asked if post-translationally buffered protein would localize to specific compartments in the cell. However, we found no enrichment in each of the subcellular localization pattern tested for this group of genes (Additional file 1: Table S4). Finally, we asked if post-translationally buffered genes are enriched for specific gene ontology groups. We found mainly enrichment of genes that are involved in the process of protein translation (Additional file 1: Table S5). Consistent with enrichment of genes involved in fundamental biological functions, we found post-translationally buffered genes to have more reported protein–protein interactions (*P* < 10^−4^) than the background genes. Taken together, these results indicated that, for genes involved in fundamental biological processes, mechanisms mediated by post-translational modifications could potentially play a role in buffering gene expression divergence present at the transcript level.

### Relaxed transcriptional regulation of buffered genes

Since protein expression levels are the main relevant quantities for gene function, we expected the presence of a conserved post-translational buffering mechanism would relax selective constraints on the transcript level. To test this hypothesis, we examined signs of constraint relaxation at the mRNA level in the context of recent human evolution. Testing for constraint relaxation at the mRNA level across primates is inappropriate in our study, since our criteria for buffered genes enriched for divergence (i.e. high variance) at the transcript level. Instead, we analyzed RNA-seq data collected from a panel of 72 human LCLs [34] and asked if the buffered genes (identified across species) have higher variation at the transcript level then the background genes. We found significantly higher mRNA variance across the panel of LCLs derived from Yoruba in Ibadan, Nigeria (YRI) for buffered genes identified between primate species (*P* < 10^−4^, Additional file 1: Table S6) (Fig. 3f, Additional file 2: Figure 3 S4). This result supported the hypothesis that selective constraint at the mRNA level is relaxed for post-translationally buffered genes. Since there is a known inverse mean-variance relationship in RNA-seq data [35, 36] (Additional file 2: Figure 3 S5), we further tested if this high variance in the buffered genes are simply an artifact driven by enrichment of genes that have lower expression levels (and therefore higher variance). Instead, we found a marginal enrichment of genes with higher expression levels; this enrichment was only found in the buffered genes identified between human and chimpanzee (i.e. not in other pairwise comparisons; Additional file 1: Table S6). This result indicated that the constraint relaxation observed in human is not simply an artifact resulting from biases introduced by differences in gene expression level. To further address potential confounding issues resulting from differences in gene expression levels between comparison groups, we performed an additional test by adjusting the background set selection to account for differences in gene expression levels (see “Methods” for details). We found the enrichment of high variance in buffered genes to remain significant after adjusting for expression levels (*P* < 10^−8^, Additional file 2: Figure 3 S6). Higher variance between individuals in buffered genes indicated that selective constraint on the mRNA levels among the YRI individuals is more relaxed for the buffered genes than for the background genes. Taken together, our results demonstrated that post-translational buffering led to a constraint relaxation at the mRNA level in recent human evolution.

## Discussion

To determine the contribution of translational regulation to gene expression level differences between humans and closely related species, we generated new data using ribosome profiling to estimate translation levels. This dataset in conjunction with the data described in Battle et al. [17] and Cenik et al. [27] provided a unique opportunity to explore recent evolution of translational regulation in humans. Through joint analysis with RNA-seq measurement of transcript levels and quantitative mass spectrometry measurement of protein levels, we provided an integrated view of divergence in gene regulation across primates. We found that divergence in translation efficiency is rare, which means that divergence between primate species at the transcript level often propagates to the level of protein translation (ribosome occupancy). This observation is in contrast to previous reports on pervasive translational buffering observed in F1 hybrids between *S. cerevisiae* and *S. paradoxus* [24, 25]. Interestingly, a report focusing on the same process in budding yeast hybrids between laboratory and wild isolate strains [23] and a follow-up reanalysis of the Artieri dataset [37] contradicts the notion of a pervasive translational buffering. Instead, their results were more in line with our observations in primates.

Translational regulation is often controlled by regulatory elements that reside in the UTR regions. Variants found in the UTR regions are therefore more likely to impact translation efficiency. Given the level of sequence divergence in the UTR regions [38], the amount of divergence in translation efficiency found between primates appears to be unexpectedly low. That being said, whether these substitutions in the UTR regions impact translational rate remains an open question. It is possible that these genetic variants, while impactful, are cryptic in the environment we tested. Further studies applying appropriate environmental perturbations could reveal species divergence in translational regulation [39]. On the other hand, we identified some inter-species divergence in translation efficiency. Interestingly, however, among the limited number of genes that show significant inter-species divergence in translation efficiency, transcriptional divergence often predicts protein level as well as (or better than) translational divergence for these genes. In other words, inter-primate divergence in translational regulation appears to have minor impact on gene expression differences at the protein level. Unfortunately, measurement noise prevented us from obtaining a precise estimate for the percentage of translational regulation that has a persistent impact on steady state protein levels. However, we were able to show that in contrast to transcriptional regulation, divergence in translation efficiency has only a minor impact on protein levels.

In contrast to gene regulation at translation, we found post-translational gene regulation to have a much broader impact on protein levels. Regulation at this layer often attenuates variation created upstream. A direct comparison between *p* values from testing effects of buffering from translational vs post-translational mechanisms clearly showed that more genes are regulated by the post-translational mechanisms. Buffering of divergence in gene expression levels has broad implications, especially in the context of evolution. For most genes, proteins often execute cellular functions. Variation in gene expression that has not reached the protein level is therefore less likely to impact organismal phenotypes. Consistent with this notion, we found evidence for relaxation of selective constraint on the mRNA levels in the HapMap YRI population for buffered genes identified between primate species. Further investigation on gene expression buffering in the context of population genetics would likely provide valuable insights on how selection might act on the regulatory variants associated with buffered genes. We found paralleled similarities between effects of post-translational buffering on gene expression divergence and effects of HSP90 chaperone action on rectifying mis-folding caused by missense mutations [40, 41]. HSP90 confers phenotypic robustness by buffering fitness impact imposed by non-synonymous mutations likely through either correcting the protein structure or facilitating the degradation process [42]. We speculate that parallel to HSP90 buffering at the structural level, post-translational buffering could confer phenotypic robustness at the gene expression level by stabilizing protein expression levels against mutations impacting transcription regulation.

We identified post-translationally buffered genes across all three pairwise species comparisons. This observation suggests that post-translational buffering is a conserved mechanism likely evolved under stabilizing selection for protein levels in primates. It remains unclear how post-translational buffering is achieved. We found enrichment of post-translational modifications among this group of genes without significant enrichment of coding substitutions. It could be that divergence in post-translational modifications instead of divergence in coding sequence led to differential turnover rates of proteins and therefore drives buffering. This interpretation provides an explanation for how post-translational buffering could be achieved between human and chimpanzee given the apparent low level of protein sequence divergence.

Post-translational buffering could be a consequence of a conserved cellular quality control system, such as endoplasmic-reticulum-associated protein degradation (ERAD) [43]. Protein quality control mechanisms are in place to ensure that proteins are properly folded and present in adequate amount to execute biological functions [44]. Adequate post-translational modifications are required for proper folding to take place. Moreover, ubiquitination is a key step in targeting mis-folded proteins to proteasome for degradations [44, 45]; misfolded proteins arising out of mutation or shortage of chaperones are labelled for degradation by ubiquitination. Consistent with the role of protein quality control mechanisms, we observed significant enrichment of reported ubiquitination sites in post-translationally buffered genes (Fig. 3e). In addition, many proteins are assembled into multi-subunit complexes with defined stoichiometry. Excess components of these complexes are targeted to proteasome for degradation [46–48]. Active degradation of excess product of translation could explain the apparent buffering of divergence at the protein level. Consistent with this notion, Chick et al. recently reported evidence supporting a stoichiometric buffering effect [18]. Moreover, Ishikawa et al. demonstrated that effects of artificial perturbation of protein stoichiometry through genetic manipulation are often buffered post-translationally [49]. Multiple protein quality control pathways could be involved in post-translational buffering. By overexpressing ribosomal proteins, Sung et al. described a nuclear protein-degradation mechanism mediated by ubiquitination in maintaining ribosomal protein stoichiometry [50]. Perhaps not coincidentally, our gene ontology analysis also found enrichment of genes that are involved in the process of protein translation for post-translationally buffered genes (Additional file 1: Table S5). Further investigation to identify factors involved in maintaining post-translational buffering would provide insights to advance our understanding of both how natural selection acts on gene regulation and how to better predict phenotypes given genetic variants that impact gene expression.

Taken together, our study provided the first integrative view on gene expression divergence across primates that allows a comparison between translational and post-translational events. We found extensive post-translational gene expression buffering that led to a stable protein level across primate species. We propose a scenario where buffering evolved under stabilizing selection of protein levels that prevents negative impacts on organismal fitness from protein level variation while allowing the transcript level to diverge for quick adaptation to environmental changes. Given the energy cost of protein translation [51], it remains puzzling to us that stabilizing selection appears to act on the post-translational level instead of the translational level. We reason that evolution of post-translational buffering is probably the more parsimonious path and speculate a *trans*-acting mechanism, involving post-translational modification enzymes, achieved gene expression buffering in a relatively short period of evolutionary time.

## Conclusions

Using ribosome profiling, we measured the impact of translational regulation on inter-species divergence between human and closely related primate species. We found divergence in translation efficiency between primate species to be relatively rare, while in contrast, we found post-translational buffering to be a major force in shaping protein expression divergence between primates. We provided evidence indicating that post-translational modifications play a role in this buffering process. Post-translational buffering appears to have allowed a relaxation in the selective constraint on transcription regulation. Taken together, these results highlighted our incomplete understanding of gene regulatory divergence between primates and have important implications for understanding human evolution.

## Methods

### Study design

The goal of this study is to evaluate the inter-species divergence in translational regulation between primates and to evaluate relative contribution of transcriptional, translational, and post-translational gene regulation to the protein level divergence between primates. This study is built on top of the previous work on quantifying inter-species divergence in transcript and protein level from human, chimpanzee, and rhesus macaque LCLs [20] and the previous work on translation QTL mapping, which collected ribosome profiling data from YRI human LCLs [17]. For evaluating inter-species divergence in protein translation, we performed ribosome profiling experiments (in one batch) for four human, four chimpanzee, and four rhesus macaque LCLs (Additional file 1: Table S1). For comparing divergence of transcriptional, translational, and post-translational regulation between primates, we leveraged data previously collected from the Khan et al. and the Battle et al. projects [17, 20]. To do so, we designed this study to include data from matching cell lines across all three datatypes (i.e. RNA-seq, ribosome profiling, and quantitative mass spectrometry). Since data from matching cell lines were collected in separate batches, to properly estimate batch effects we also included additional cell lines from each batch (Additional file 1: Table S7). Details on how batch effects were adjusted and how differential expression tests were done can be found in respective sections. While these adjustments were necessary to ensure accurate identification of divergent genes, the main conclusions of the paper are robust against the exact method of choice.

### Cell culture and ribosome profiling

Ribosome profiling data were collected from LCLs derived from five chimpanzee (*Pan troglodytes*) individuals (New Iberia Research Center: Min 18358, Min 18359; Coriell/IPBIR: NS03659, NS04973, Arizona State University: Pt91; all Epstein-Barr virus (EBV)-transformed), five rhesus macaque (*Macaca mulatta*) individuals (Harvard Medical School, NEPRC: 150–99, R181–96, R249–97, 265–95, R290–96; all rhesus Herpesvirus papio transformed), and four human individuals (Coriell: GM19127, GM19137, GM19144, GM19147; all EBV-transformed). Cell lines were cultured at 37 °C with 5% CO_2_ in RPMI media with 15% FBS. The media were further supplemented with 2 mM L-glutamate, 100 IU/mL penicillin, and 100 μg/mL streptomycin. Before pelleting the cells for lysate preparation, we did not incubate the culture with cycloheximide. We avoided cycloheximide treatment because of its known potential in introducing biases at certain codons [52]. Ribosome profiling experiments were performed using ARTseq™ Ribosome Profiling kit for mammalian cells (RPHMR12126) following vendor’s instructions. Briefly, cell lysates were prepared by disrupting flash frozen pellets of 30–50 million live cells through repeated pipetting in 1 mL cold lysis buffer on ice. Monosome isolation was performed using Sephacryl S400 spin columns (GE; 27–5140-01) on a tabletop centrifuge. Ribosomal RNA depletion was carried out by using Ribo-Zero Magnetic Kits (Epicentre; MRZH11124). Ribosome footprint complementary DNA libraries were PCR amplified (12–15 thermo-cycles) and barcoded using ScriptMiner Index PCR Primers (Epicentre; SMIP2124). Indexed libraries were subsequently pooled together and then sequenced on an Illumina HiSeq 2500.

### Data processing

#### Preprocessing, mapping, and counting

Since ribosome protected fragments are relatively short (~ 30 nt), a typical 50-cycle Illumina sequencing run will read into the adaptor sequence. Before aligning reads to the genome, we first removed adaptor sequence using FASTX-Toolkit. We also trimmed the 5′ most nucleotide from each read, as it has been reported that this nucleotide is often an artifact resulted from non-templated addition from the reverse transcription step during library construction [21]. Ribosome profiling strategy enriches for sequence reads derived from rRNA, transfer RNA (tRNA), and, to a lesser extent, small nuclear RNA (snoRNA). Because our goal was to quantify the level of protein translation, to facilitate downstream analyses, we first removed sequencing reads mapped to a reference FASTA file composed of human rRNA, tRNA, and snoRNA sequences. The unmapped reads resulted from this filtering step were kept and then aligned to their respective reference genome (i.e. hg19 for human, Pantro3 for chimpanzee, and RheMac2 for rhesus macaque) using BWA [53]. The mapping procedure allowed a maximum of two mismatches and retained only uniquely mapped reads (see Khan et al. [20] for the exact BWA options used). Alignments with quality scores < 10 were filtered out using SAMtools [54]. Levels of protein translation were estimated by counting the number of ribosome profiling reads aligning to each gene using BEDTools [55]. Importantly, for inter-species comparison, we considered only reads intersecting exons that are orthologous between species (used in [29]; Additional file 3). Junction reads spanning across exons were excluded from this analysis.

#### Data quality assessment

##### Codon periodicity

Ribosome footprints are enriched at coding exons in a pattern that reflects the mechanism of protein translation [21]. We use this pattern (see Fig. 1a for examples) as a quality check to evaluate each ribosome profiling experiment. To compute a quantitative metric, we aggregated data across all conserved translation initiation sites on the plus strand of the human genome. To obtain annotations for conserved translation initiation sites, we first downloaded coding exon annotations from UCSC genome browser [56] and used a 100-bp window flanking the first position of the first coding exon for each gene as a candidate region. We then computed average PhastCons scores [57] for each candidate region and set a conservation cut-off at an average PhastCons score of 0.9 to define the regions flanking conserved translation initiation sites in human. Using this approach, we defined 668 conserved translation initiation sites for human. For chimpanzee and rhesus macaque, translation initiation site annotations were separately converted from the human annotations using liftOver [58]. To compute aggregate enrichment at each position relative to the initiation site, we used BEDTools to identify RPF overlapping the 100-bp conserved initiation site windows and took only the 5′ most position of each RPF to represent the position of the ribosome (for Fig. 1a, we shifted the relative coordinates to center the enrichment pattern at the P site of a ribosome). Relative enrichment at each position was then computed by summing the number of reads for each position and then dividing by the total number of reads that fall into the 100-bp windows across conserved translation initiation sites.

##### Principal components analysis and potential confounders

Singular value decomposition was performed on centered and scaled ribosome profiling data using the prcomp() function in the stats package of R environment for statistical computing. To evaluate impact of potential confounders on variation between samples, we tested for associations between each principal component (PC) and potential confounders (Additional file 2: Figure 1 S4). For quantitative confounders, we computed Pearson’s correlation coefficients; for qualitative confounders, we used analysis of variance.

#### Data transformation

We focused our analysis on genes that were reliably quantified across all three species. To do this, we kept only genes that have at least one sequencing read aligned to in at least three out of four individuals of each species. This filtering resulted in a quantification matrix of 9364 detectable genes (Additional file 4). To test for differential expression using a linear modeling framework, we transformed the raw counts to TMM-normalized log_2_ counts per million (CPM) [59]. We then account for variations in orthologous gene lengths across species by converting log_2_CPM to log_2_ reads per kilobase per million (RPKM) using species-specific gene lengths. To adjust for heteroscedasticity in sequencing data for linear modeling, we also estimated observational-level weights using voom [36].

Since ribosome footprints are shorter than the typical RNA sequencing reads, additional inter-species mappability differences could arise specifically in ribosome profiling data. To evaluate the potential impact of mappability differences on differential expression analysis, we performed an alternative data transformation to account for inter-species mappability differences. To do so, we first identified mappable regions for each orthologous gene for each species and then used species–specific mappable gene lengths for converting CPM to RPKM. To compute species-specific mappable gene lengths, we first generated in silico synthetic footprints of 29 nt (i.e. the median footprint length) tiling all orthologous exons at a single base increment for each species. We then identify mappable regions for each species by mapping the synthetic footprints back to the respective genome. We evaluate the impact of inter-species mappability differences on results of differential expression analysis by the coefficient of determination computed between test results (i.e. fold change and *p* value) obtained with and without adjusting for mappability differences.

For genes that were not reliably quantified across all three species, we further categorized genes that were expressed in at least one species and genes that were only expressed in one species. For a gene to qualify as expressed in (at least) one species, we required an average within species expression level to be greater than the first quartile (i.e. 25%, RPKM = 6.05) of the detectable genes (Additional file 4) and a minimum of one sequencing read aligned to the gene in at least three individuals of the species. For a gene to qualify as expressed in only one species, we further required the expression level to only be quantifiable in at most one individual from each of the other two species (Additional file 6).

For joint analyses across different data types, we included additional ribosome profiling data to have quantifications on a matching set of 15 individuals that we had previously collected RNA-seq data and quantitative mass spectrometry data [20]. Since ribosome profiling data from additional human individuals were collected across multiple batches as a part of an earlier study [17], to adjust for batch effects, we first included multiple individuals from each relevant batch to better estimate the effects introduced by batch differences. Data from individuals other than the 15 cell lines matching previous RNA-seq and mass spectrometry data collection (Additional file 1: Table S7) were only included for batch effect adjustment and were excluded from the downstream joint analyses. Before adjusting for batch effects, we filtered out genes that were not sufficiently quantified for estimating batch effects by requiring a minimum of quantification in two individuals for each batch. Filtered data were then TMM normalized and transformed to log_2_RPKM. We used Combat with a parametric prior to adjust for batch effects [30]. We then extracted batch-effect-adjusted data from the 15 individuals of interest for subsequent differential expression analyses. The same normalization and batch effect adjustment procedures were performed on the RNA-seq dataset using its respective batch information. For protein data, we first filtered out genes that were not expressed at the protein level by requiring available SILAC ratios from at least three individuals from each species for each gene. We then normalized the filtered data by centering SILAC ratios quantified for each cell line at its respective trimmed mean (i.e. shifting the entire distribution for each individual sample to get a trimmed mean of zero). When calculating trimmed mean for each individual, we excluded the top and bottom 30% of genes. Finally, for joint analyses across different data types, we only included genes that were quantified across all three data types (Additional file 7).

### Differential expression test

Differential expression tests were computed using limma [60, 61] R Bioconductor package.

For testing differences in levels of protein translation between species, we fitted log_2_ transformed, TMM normalized ribosome profiling data to a fixed effects model (species effect) with voom weights [36]. For each gene, the species coefficient (effectively log_2_ ratio between species) is tested against the null hypothesis that the coefficient is equal to zero using empirical Bayes moderated t-statistics. To account for multiple testing, nominal *p* values were adjusted using Bonferroni correction to get estimates of FWER; false discovery rates (FDR) estimated using qvalue [62] were also included in the supplemental files. While we included this information to help readers interpreting the results, it should be noted that in some instances, the *p* value distributions are not ideal for applying qvalue() function, and the resulting FDRs should therefore be interpreted with precaution. For analyses assessing sex effect in ribosome profiling data, we adjusted for sex effect on log2 transformed and TMM normalized ribosome profiling data using Combat with parametric prior [30].

When testing for differences in translation efficiency between species, we jointly modeled ribosome profiling and RNA-seq data with a fixed effects model including an interaction term. The interaction coefficient estimates datatype dependent species difference (i.e. species difference in translation efficiency); thus, it identifies differences in translation that cannot be accounted for by differences in transcript expression and vice versa. More specifically, for each gene, let *E*_*ij*_ be expression level of data type *j* from individual *i*, *S*_*ij*_ be the indicator variable for species label, and *T*_*ij*_ be the indicator variable for data type label. We fitted the following model:

$$ {E}_{ij}=\mu +{\beta}_1{S}_{ij}+{\beta}_2{T}_{ij}+{\beta}_3{S}_{ij}{T}_{ij}+{\upvarepsilon}_{ij} $$

We fitted log_2_ transformed, TMM normalized ribosome profiling data and RNA-seq data jointly with voom weights. For each gene, we used empirical Bayes moderated t-statistics to test the contrast coefficient for the interaction term (i.e. β_3_, which is effectively log_2_ ratio of translation efficiency between species) against the null hypothesis that the coefficient is equal to zero.

When testing for gene expression buffering, we first regressed out downstream effects using linear models and then tested for species effects on the residuals using the same limma framework described above. For testing translational buffering, we defined transcript level (RNA-seq) as the upstream trait and level of protein translation (ribosome profiling) as the downstream trait. For testing post-translational buffering, we defined level of protein translation (ribosome profiling) as the upstream trait and protein levels (quantitative mass spectrometry) as the downstream trait. For each comparison, we only considered a pair of species (i.e. instead of fitting all three species together). We first modeled the upstream trait (dependent variable) with the downstream trait as a fixed-effect predictor. More specifically, for each gene, let *U*_*i*_ be expression level of an upstream trait from individual *i*, *D*_*i*_ be expression level of a downstream trait, *S*_*i*_ be the indicator variable for species label. We first fitted the following model:

$$ {U}_i=\mu +{\beta}_1{D}_i+{\upvarepsilon}_i $$

We then took the residual from the first model fit (i.e. ε_*i*_) and further fitted a second model on the residual with species as a fixed-effect predictor:

$$ {\upvarepsilon}_i={\mu}^{\prime }+{\beta}_2{S}_i+\upvarepsilon {\prime}_i $$

The estimated species coefficients (i.e. *β*_2_) from the second model fit were used to quantify residual divergence between species for the upstream molecular trait after accounted for the divergence in the downstream molecular trait (i.e. effect size of buffering).

### Enrichment analysis

Enrichment of different features in post-translationally buffered genes were evaluated by testing Pearson’s product moment correlation coefficients between the features of interest (either a continuous or binary distribution across genes) and significance of buffering (i.e. -log_10_(*p* value) from post-translational buffering tests). Unless otherwise specified, all tests were two-sided and the sign of correlation coefficient indicated the direction of enrichment. For Gene Ontology analysis, we adjusted for multiple testing using the Benjamini–Hochberg FDR.

Annotations for post-translational modifications were downloaded from PhosphoSitePlus [63] on 5 August 2016. Subcellular location and Gene Ontology annotations were downloaded from UniProt [64] (last updated on 6 July 2016). Protein–protein interaction data were downloaded from BioGRID v3.4 [65] (last updated on 26 July 2016). Pairwise inter-species Ka, Ks values, and other gene features (such as gene length and GC content) were downloaded from Ensembl [66] for genome build hg19. Finally, for genes that have multiple isoforms, we used the median value among isoforms for each feature of interest to test for enrichment.

## Additional files

Additional file 1: Supplementary tables. (PDF 53 kb)

Additional file 2: Supplementary figures. (PDF 13403 kb)

Additional file 3: Genome coordinates of primate orthologous exons. A .txt file listing genome coordinates for human (hg19), chimpanzee (Pantro3), and rhesus macaque (rheMac2) for orthologous exons used in this study. (TXT 20206 kb)

Additional file 4: Ribosome profiling count Table. A .csv file listing raw ribosome profiling counts for genes that are quantifiable across all three species. (CSV 565 kb)

Additional file 5: Inter-species divergence in protein translation. A .csv file listing results from testing for differences in levels of protein translation between species for genes that are quantifiable in all three species. HvC.beta: coefficient estimated from a contrast comparing between human and chimpanzee (effectively log_2_ ratio of levels of protein translation between the two species). HvC.p.value: nominal *p* values derived from t tests. HvC.FDR: false discovery rate adjusted from nominal *p* value. HvC.FWER: family-wise error rate adjusted from nominal *p* value. (CSV 1507 kb)

Additional file 6: Species-specific protein translation. A .csv table of raw ribosome profiling counts listing genes that are quantifiable in at least one species (see details on criteria in “Methods”). Columns of Boolean labels indicate whether or not a gene is expressed in a species and whether a gene is only expressed in that species. (CSV 103 kb)

Additional file 7: Transformed ribosome profiling, RNA-seq, and quantitative mass spectrometry data for genes that are quantifiable in all three species across all three data types. A total of six R objects are included in this .RData file. Ribo.expressed.data: TMM normalized log_2_RPKM values of ribosome profiling data, ribo.expressed.weights: corresponding voom weights for ribosome profiling data, ribo.expressed.ref: TMM normalized log_2_RPKM values of ribosome profiling data for the reference cell line (GM19238), RNA.expressed.data: TMM normalized log_2_RPKM values of RNA-seq data, RNA.expressed.weights: corresponding voom weights for RNA-seq data, RNA.expressed.ref: TMM normalized log_2_RPKM values of RNA-seq data for the reference cell line (GM19238), protein.expressed.data: trimmed mean centered SILAC ratios for quantitative mass spectrometry data. (RDATA 1942 kb)

Additional file 8: Inter-species divergence in translation efficiency. A .csv file listing results from testing for differences in translation efficiency between species for genes that are quantifiable in all three species across all three data types. Column names follow the same convention as Additional file 5. (CSV 606 kb)

Additional file 9: Translational gene expression buffering. A .csv file listing results from testing for translational gene expression buffering between species for genes that are quantifiable in all three species across all three data types. Column names follow the same convention as Additional file 5. (CSV 600 kb)

Additional file 10: Post-translational gene expression buffering. A .csv file listing results from testing for post-translational gene expression buffering between species for genes that are quantifiable in all three species across all three data types. Column names follow the same convention as Additional file 5. (CSV 607 kb)
