# Supplementary material for: Post-translational buffering leads to convergent protein expression levels between primates
Source: Genome Biol. 2018 Jun 27;19:83. doi: 10.1186/s13059-018-1451-z (PMC6020354; doi:10.1186/s13059-018-1451-z)
Supplement: Supplementary file 1 — Supplementary tables. (PDF 53 kb) [file 13059_2018_1451_MOESM1_ESM.pdf]

Table S1 Ribosome profiling data generated for studying species divergence in levels of protein translation.

ID	mapped count	proportion > Q30	species
**C18358**	7,393,603	0.971	chimpanzee
**C18359**	15,478,039	0.970	chimpanzee
**C3659**	20,284,521	0.972	chimpanzee
**CPt91**	10,079,028	0.970	chimpanzee
**R181-96**	12,599,203	0.979	rhesus macaque
**R249-97**	12,040,159	0.974	rhesus macaque
**R265-95**	12,063,859	0.979	rhesus macaque
**R290-96**	10,491,523	0.977	rhesus macaque
**H19127**	8,984,640	0.977	human
**H19137**	9,754,058	0.977	human
**H19144**	12,835,528	0.974	human
**H19147**	14,949,479	0.977	human

* mapped count: total number of uniquely mapped reads for each sample after removing rRNA, tRNA and snoRNA reads.

* proportion > Q30: proportion of sequencing reads that have a Phred quality score greater than 30.

Table S2 Enrichment of nonsynonymous substitutions in post-translationally buffered genes.

	**HvC.cor**	**HvC.p.value**	**RvC.cor**	**RvC.p.value**	**RvH.cor**	**RvH.p.value**
**Ka**	-	0.93	-	0.17	+	0.63
**Ka/Ks**	-	0.87	-	0.14	+	0.68

* HvC: human vs. chimpanzee, RvC: rhesus macaque vs. chimpanzee, RvH: rhesus macaque vs. human.

*cor: direction of correlation, + enrichment, - depletion.

*p.value: probability of observing the correlation when there is no enrichment in the feature of interest in post-translationally buffered gene identified between each pair of species.

Table S3 Enrichment of protein modifications in post-translationally buffered genes.

PTM type	HvC.cor	HvC.p.value	RvC.cor	RvC.p.value	RvH.cor	RvH.p.value
**Ubiquitination**	+	2.20E-16	+	6.66E-16	+	2.20E-16
**Phosphorylation**	+	2.51E-08	+	1.29E-02	+	4.40E-06
**Acetylation**	+	2.20E-16	+	7.74E-08	+	2.39E-10
**Methylation**	+	4.76E-05	+	1.34E-02	+	1.84E-04
**Sumoylation**	+	3.74E-07	+	1.14E-03	+	2.07E-04
**O-GalNAc**	-	3.76E-01	-	2.99E-01	+	7.98E-01
**O-GlcNAc**	-	9.86E-01	+	6.18E-01	+	4.93E-02

* HvC: human vs. chimpanzee, RvC: rhesus macaque vs. chimpanzee, RvH: rhesus macaque vs. human.

*cor: direction of correlation, + enrichment, - depletion.

*p.value: probability of observing the correlation when there is no enrichment in the feature of interest in post-translationally buffered gene identified between each pair of species.

Table S4 Enrichment of sub-cellular locations for post-translationally buffered genes.

SubCellLoc	NO. of genes associated	HvC.cor	RvC.cor	HvR.cor	HvC.p.value	RvC.p.value	HvR.p.value
**Cytoplasm**	1393|1773	-	+	+	3.53E-01	6.12E-01	9.61E-01
**Endoplasmic**	263|2903	-	+	+	1.55E-01	2.04E-01	7.06E-01
**Golgi**	222|2944	-	-	-	2.16E-03	2.28E-01	1.55E-01
**Cytosol**	136|3030	-	-	-	1.96E-01	1.50E-02	1.87E-01
**Nucleus**	1239|1927	+	+	+	1.32E-05	3.30E-01	5.34E-01
**Lysosome**	64|3102	-	-	-	2.59E-01	3.19E-01	7.65E-01
**Mitochondrion**	426|2740	-	-	-	7.03E-03	2.30E-04	7.20E-02
**Peroxisome**	26|3140	-	-	-	5.92E-01	4.41E-01	9.96E-01
**Membrane**	232|2934	-	-	+	4.24E-02	7.37E-01	2.88E-01
**Endosome**	112|3054	-	-	-	2.01E-02	7.26E-02	1.06E-02
**Secreted**	55|3111	-	-	+	6.67E-01	5.81E-01	1.61E-01
**P-body**	24|3142	-	+	+	9.73E-01	2.46E-01	4.84E-01

* HvC: human vs. chimpanzee, RvC: rhesus macaque vs. chimpanzee, HvR: rhesus macaque vs. human.

*cor: direction of correlation, + enrichment, - depletion.

*p.value: probability of observing the correlation when there is no enrichment in the feature of interest in post-translationally buffered gene identified between each pair of species.

Table S5 Enrichment of functional categories for post-translationally buffered genes.

GO.ID	Term	NO. of genes associated	HvC.fdr	RvC.fdr	HvR.fdr
**GO:0000184**	nuclear-transcribed mRNA catabolic process & nonsense-mediated decay	51|3115	2.29E-13	8.13E-13	1.52E-13
**GO:0003723**	RNA binding	192|2974	5.09E-07	2.59E-03	1.08E-05
**GO:0003735**	structural constituent of ribosome	90|3076	2.11E-09	2.09E-03	3.73E-10
**GO:0006364**	rRNA processing	107|3059	2.29E-13	8.80E-06	1.52E-13
**GO:0006412**	translation	91|3075	9.45E-08	3.77E-05	3.92E-10
**GO:0006413**	translational initiation	59|3107	2.29E-13	2.29E-13	1.52E-13
**GO:0006614**	SRP-dependent cotranslational protein targeting to membrane	40|3126	2.29E-13	2.29E-13	1.52E-13
**GO:0015935**	small ribosomal subunit	5|3161	5.08E-05	3.19E-06	3.07E-03
**GO:0019083**	viral transcription	54|3112	2.29E-13	2.29E-13	1.52E-13
**GO:0022625**	cytosolic large ribosomal subunit	23|3143	2.29E-13	1.26E-04	1.52E-13
**GO:0022627**	cytosolic small ribosomal subunit	13|3153	1.46E-04	2.29E-13	1.52E-13
**GO:0030529**	ribonucleoprotein complex	56|3110	6.29E-05	7.29E-03	1.95E-03

* HvC: human vs. chimpanzee, RvC: rhesus macaque vs. chimpanzee, HvR: rhesus macaque vs. human.

*fdr: False discovery rates (Benjamini-Hochberg) of enrichment in the feature of interest in post-translationally buffered gene identified between each pair of species * Only GO terms that pass a 1% false discovery rate cutoff are presented.

* All associations reported in this table are positive correlations (i.e. enrichment)

Table S6 Post-translationally buffered genes have more variable transcript expression levels in an YRI population.

feature	HvC.cor	HvC.p.value	RvC.cor	RvC.p.value	HvR.cor	HvR.p.value
**RNA.mean**	+	2.02E-03	+	5.76E-01	-	7.29E-01
**RNA.sd**	+	4.47E-07	+	5.44E-05	+	1.50E-07
**RNA.TMM.mean**	+	2.02E-03	+	5.76E-01	-	7.29E-01
**RNA.TMM.sd**	+	1.17E-06	+	1.05E-04	+	1.29E-06
**RNA.QN.mean**	+	1.82E-03	+	5.70E-01	-	7.38E-01
**RNA.QN.sd**	+	1.72E-08	+	1.18E-05	+	2.40E-08

* HvC: human vs. chimpanzee, RvC: rhesus macaque vs. chimpanzee, HvR: rhesus macaque vs. human.

*cor: direction of correlation, + enrichment, - depletion.

*p.value: probability of observing the correlation when there is no enrichment in the feature of interest in post-translationally buffered gene identified between each pair of species.

*mean: average transcript levels across YRI individuals

*sd: standard deviation of transcript levels across YRI individuals

*TMM: trimmed-mean normalized

*QN: quantile normalized

Table S7 Additional ribosome profiling data used for batch effect adjustment and for joint analysis with RNA seq and quantitative mass spec data.

ID	mapped count	proportion > Q30	species	batch
**C4973**	14,640,312	0.979	chimpanzee	mm8
**H18505**	11,952,124	0.919	human	mm1
**H18507**	7,864,959	0.947	human	mm1
**H18516**	5,009,693	0.923	human	mm1
**H19193**	10,081,244	0.919	human	mm1
**H19204**	15,820,298	0.975	human	mm2
**H19238**	8,941,143	0.984	human	mm7
**R150-99**	14,008,661	0.978	rhesus macaque	mm8
**H18909**	11,532,097	0.973	human	mm2
**H19099**	13,030,793	0.973	human	mm2
**H19143**	8,312,531	0.973	human	mm2
**H19201**	18,731,551	0.986	human	mm7
**H19210**	12,817,715	0.986	human	mm7
**H19223**	10,814,930	0.985	human	mm7

* mapped count: total number of uniquely mapped reads for each sample after removing rRNA, tRNA and snoRNA reads.

* proportion > Q30: proportion of sequencing reads that have a Phred quality score greater than 30.
