# Supplementary material for: Post-translational buffering leads to convergent protein expression levels between primates
Source: Genome Biol. 2018 Jun 27;19:83. doi: 10.1186/s13059-018-1451-z (PMC6020354; doi:10.1186/s13059-018-1451-z)
Supplement: Supplementary file 2 — Supplementary figures. (PDF 13403 kb) [file 13059_2018_1451_MOESM2_ESM.pdf]

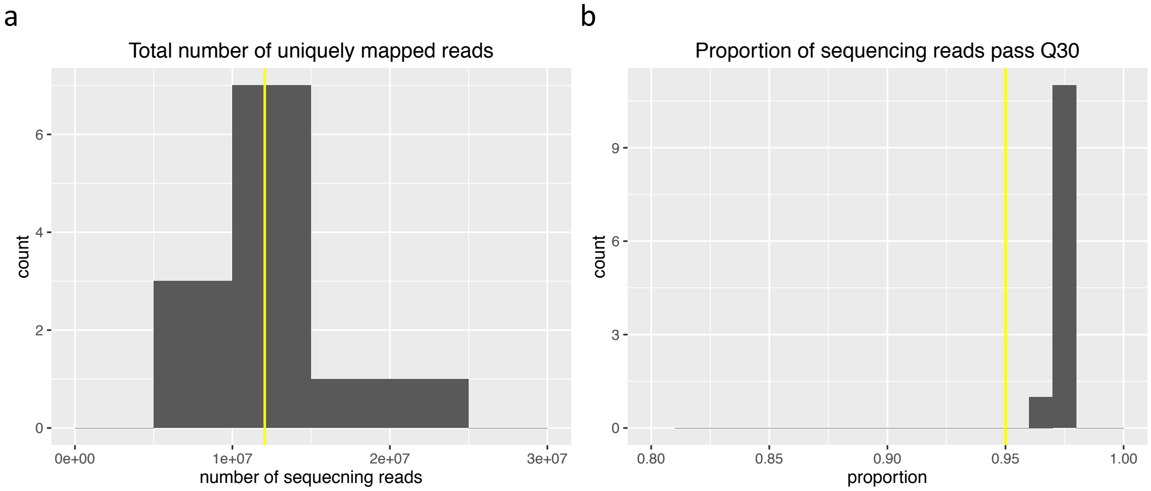

Figure 1 S1 Sequencing coverage and quality. (a) A histogram summarizing the total number of uniquely mapped sequencing reads for each sample. The yellow vertical line marks the median. (b) A histogram summarizing the proportion of sequencing reads that have Phred quality scores greater than 30 for each sample. For all samples, more than 95% (vertical yellow line) of sequencing reads are of high quality (i.e. Phred score > 30).

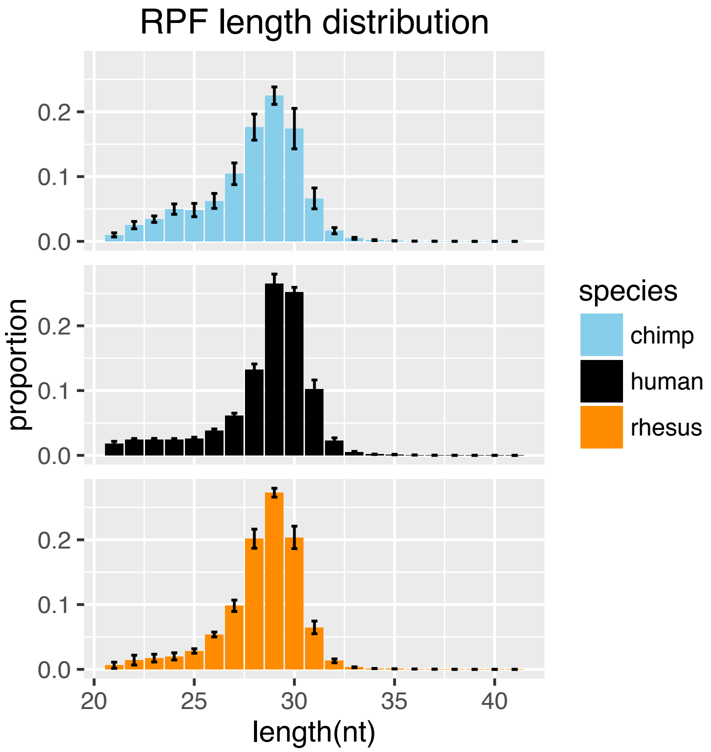

Figure 1 S2 Length distribution of RPF is consistent with the expected size of ribosome footprints. Bar plots showing fragment length distribution of RPF. To control for differences in sequencing depth between samples, number of RPF reads of each fragment size is converted to proportion over total number of reads for each sample. Bar height represents mean proportion ± standard error estimated from biological replicates for each species.

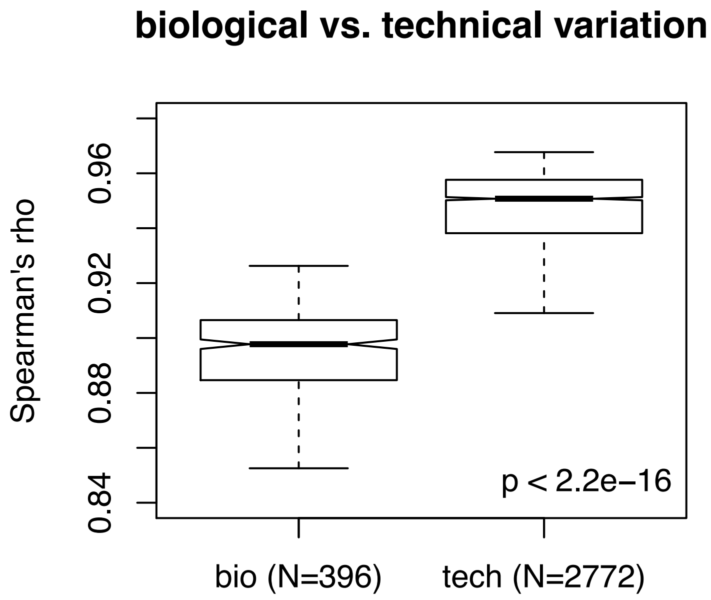

Figure 1 S3 Technical variation is significantly smaller than biological variation. Boxplots comparing correlations (Spearman's rho) between technical replicates to correlations between biological replicates. Note that a higher correlation between replicates indicates less variation. N: the number of Spearman's rho summarized, bio: pairwise correlations between data generated from different samples of the same species that were sequenced in the same lane, tech: pairwise correlations between data generated from the same sample that were sequenced in different lanes.

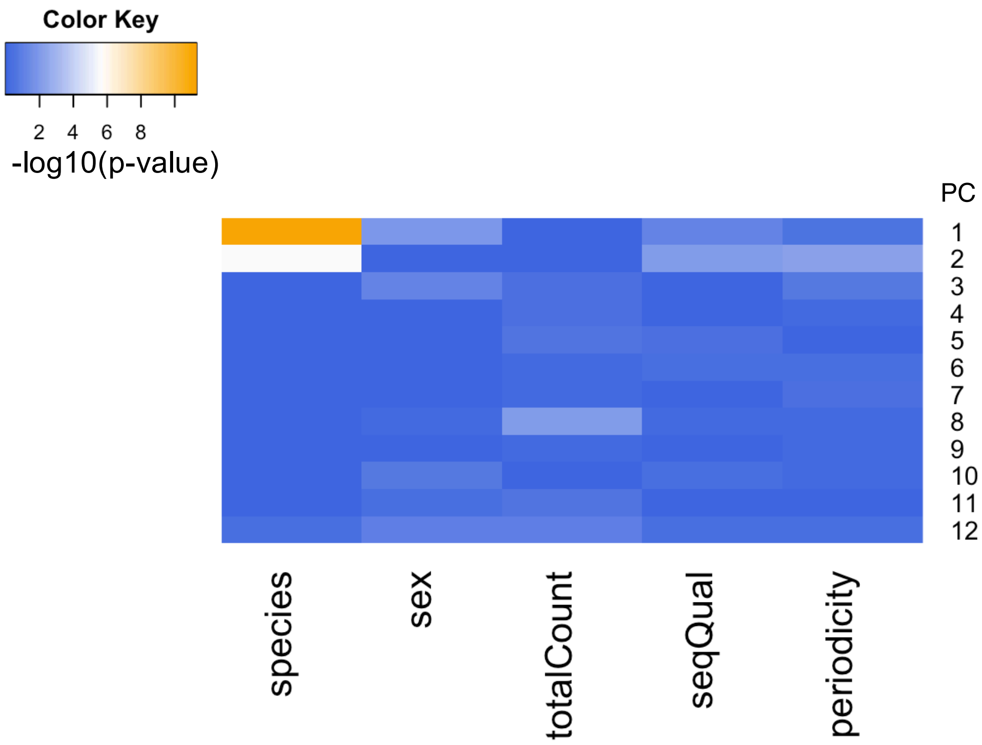

Figure 1 S4 Correlations between major signals in the ribosome profiling data and various biological or technical variables. A heatmap of $-\log_{10}$(p-values) showing significance level for correlations between major principal components (PC) and each of the variables. totalCount: number of uniquely mapped reads from each sample. seqQual: proportion of sequencing reads pass a Phred quality score cutoff of 30. periodicity: strength of subcodon periodicity of each sample (see Methods).

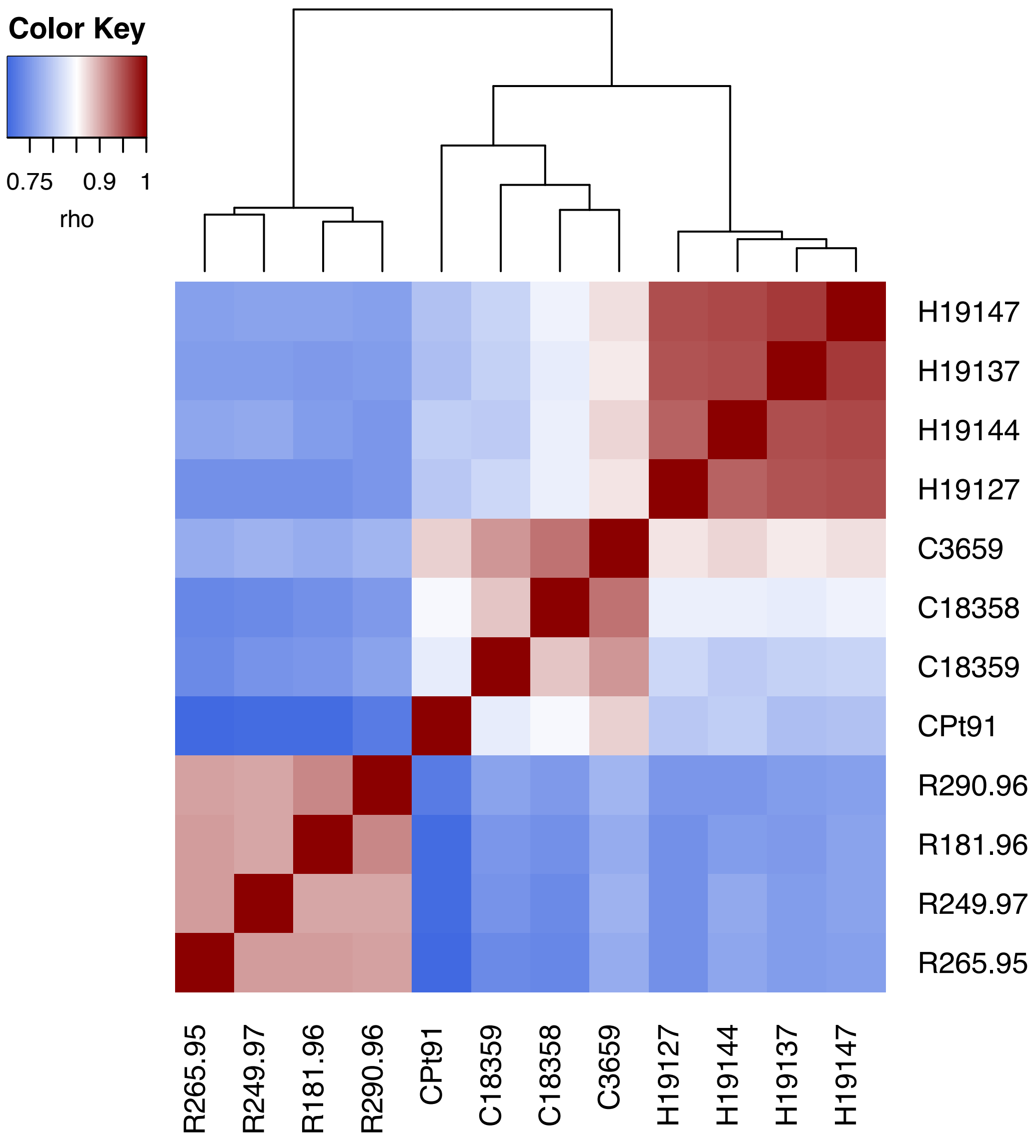

Figure 1 S5 The major signal in the ribosome profiling data reflects species differences. A heatmap of pairwise correlations (Spearman's rho) for all samples. Branch length of the dendrogram on top reflects Euclidean distance between columns of correlations.

a

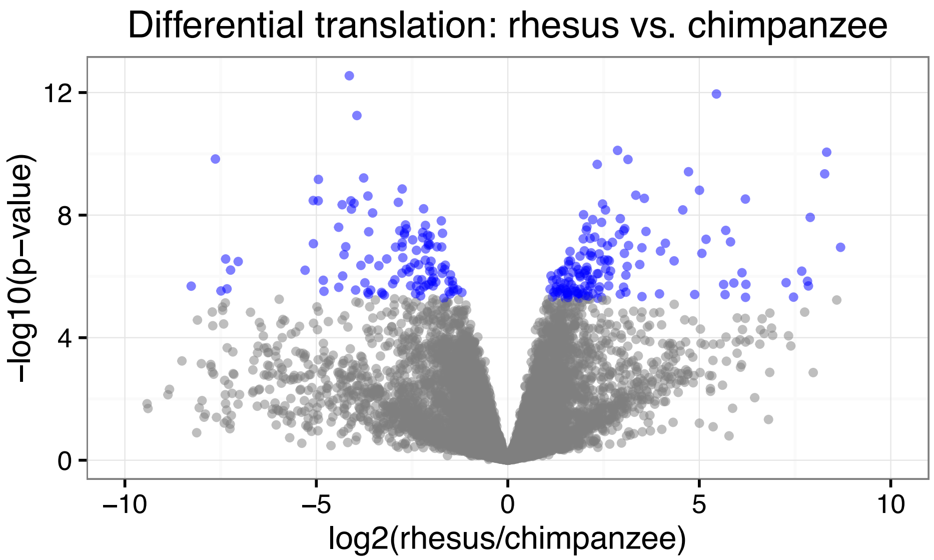

b

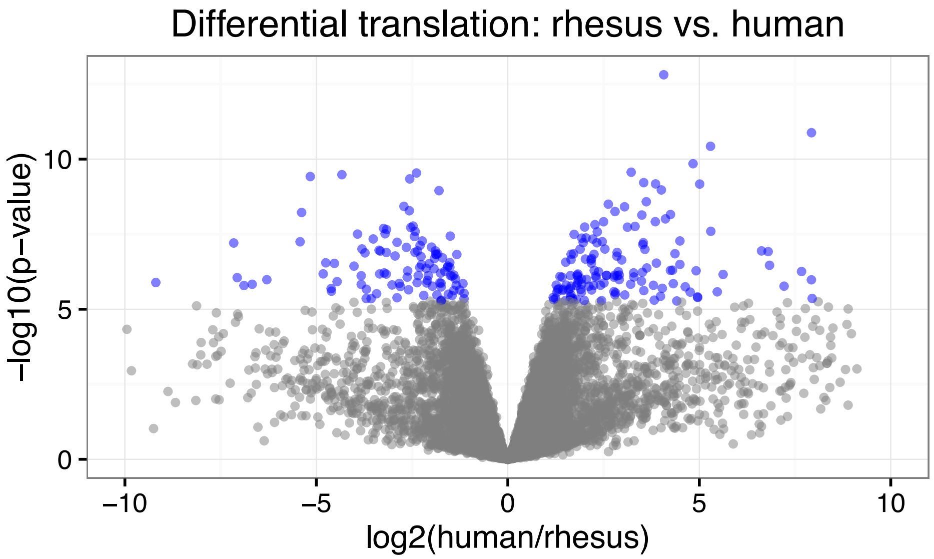

Figure 1 S6 Divergence in level of protein translation between species. Each data point represents a gene: position along the x-axis indicates log_2 ratio of ribosome occupancy level between two species, position along the y-axis indicates significance level, and the color of each data point indicates whether the gene is significantly diverged between species at a significance cutoff of FWER 0.05 (blue: significant, grey: not significant). (a) rhesus macaque vs. chimpanzee. (b) rhesus macaque vs. human.

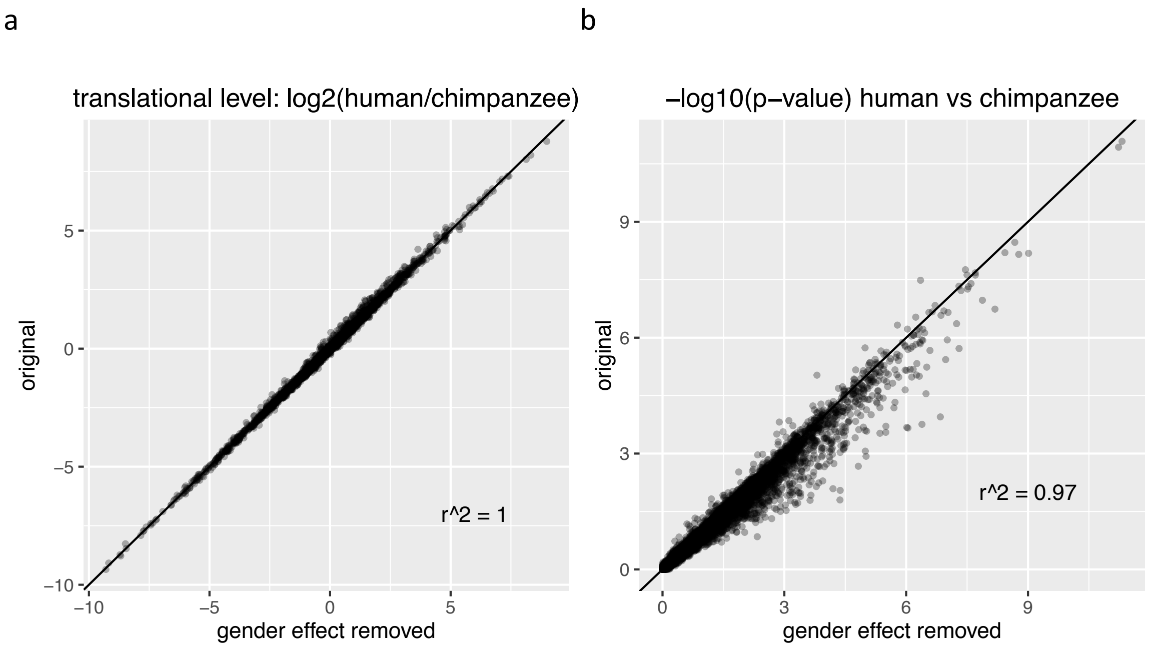

Figure 1 S7 Sex effect has little to no impact on results from differential expression (translation) tests between species using the ribosome profiling data. (a) A scatter plot comparing between effect size estimates derived from data with and without gender effect. Each data point represents a gene, position along each axis indicates $\log_2$ ratio of ribosome occupancy level between human and chimpanzee. (b) A scatter plot comparing between p-values derived from differential expression tests done on data with and without gender effect. Each data point represents a gene, position along each axis indicates $-\log_{10}$(p-values) computed from tests for differences in level of protein translation between human and chimpanzee.

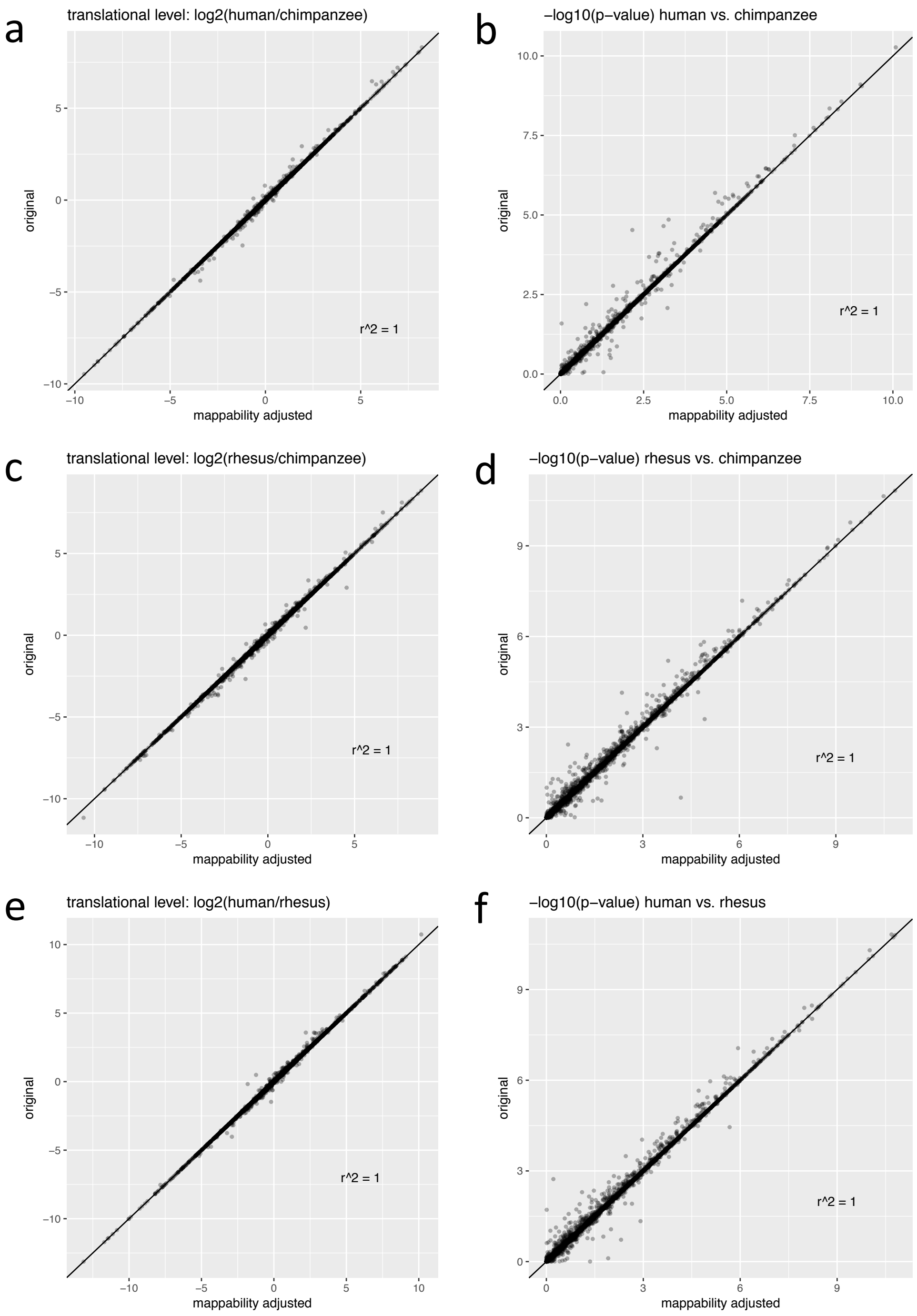

Figure 1 S8 Inter-species mappability differences have little to no impact on results from inter-species differential expression (translation) tests using ribosome profiling data. (a, c, e) Scatter plots comparing between effect size estimates derived from data with and without adjusting for mappability differences. Each data point represents a gene, position along each axis

indicates log_2 ratio of ribosome occupancy level between the species specified. (b, d, f) Scatter plots comparing between p-values derived from differential expression tests done on data with and without adjusting for mappability differences. Each data point represents a gene, position along each axis indicates $-log_{10}$(p-values) computed from tests for differences in level of protein translation between the species specified.

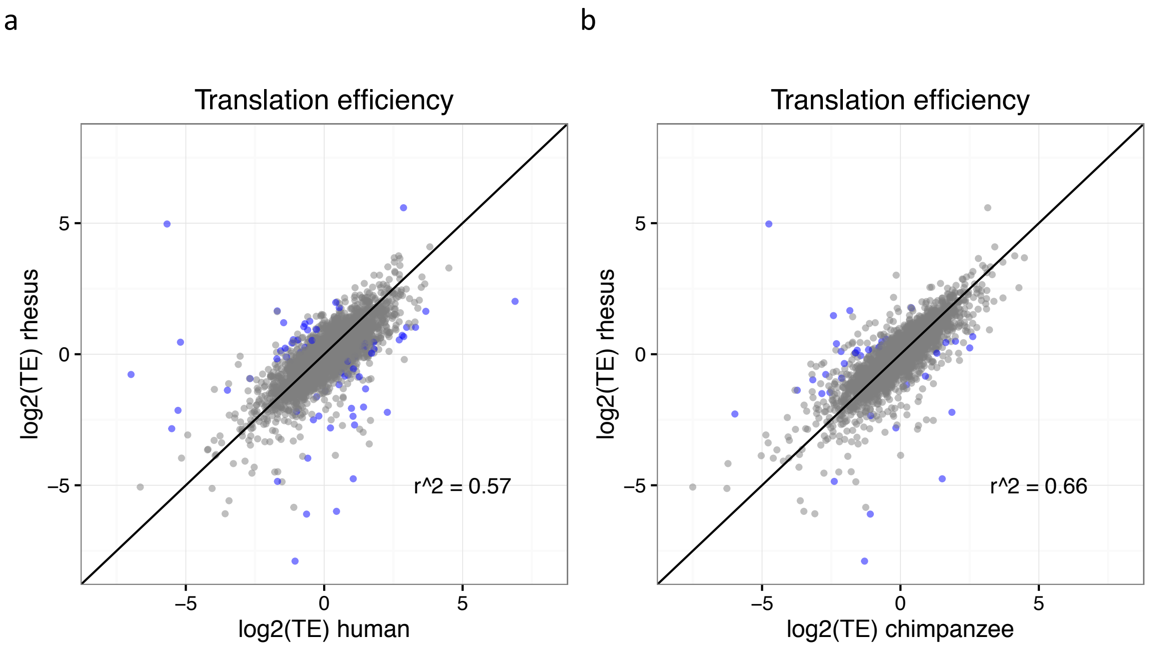

Figure 2 S1 Inter-species divergence in translation efficiency. Scatter plots of translation efficiency (TE) comparing between (a) human and rhesus macaque (b) chimpanzee and rhesus macaque. Each data point represents a gene, position along each axis indicates $\log_2$ translation efficiency of each species, and the color of each data point indicates whether the gene is significantly diverged between species at a significance cutoff of FWER 0.05 (blue: significant, grey: not significant).

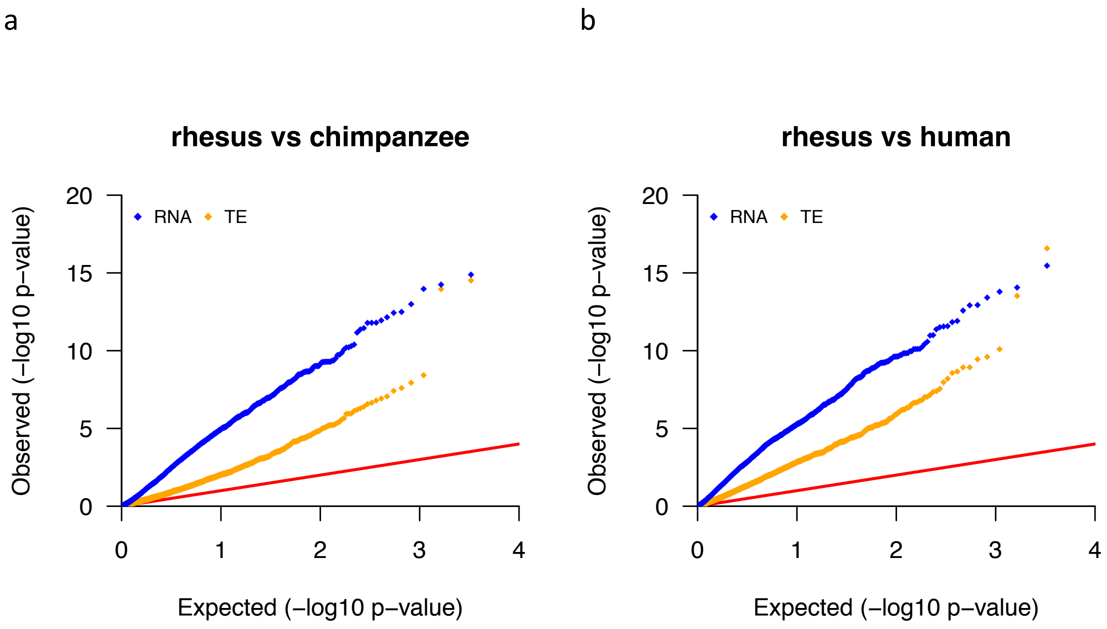

Figure 2 S2 Significant divergence between primate species occurs more frequently at the transcript level than at the level of translation efficiency. Quantile-qunatile plot of $-\log_{10}$(p-values) derived from testing for inter-species divergence for each trait of interest (RNA: transcript level, TE: translation efficiency). (a) Divergence between rhesus macaque and chimpanzee. (b) Divergence between rhesus macaque and human. For each molecular trait, observed p-value (y-axis) is plotted against the null expectation (i.e. uniform distribution of p-values) (x-axis). The red line marks the expected results from a scenario where no divergence between species is observed.

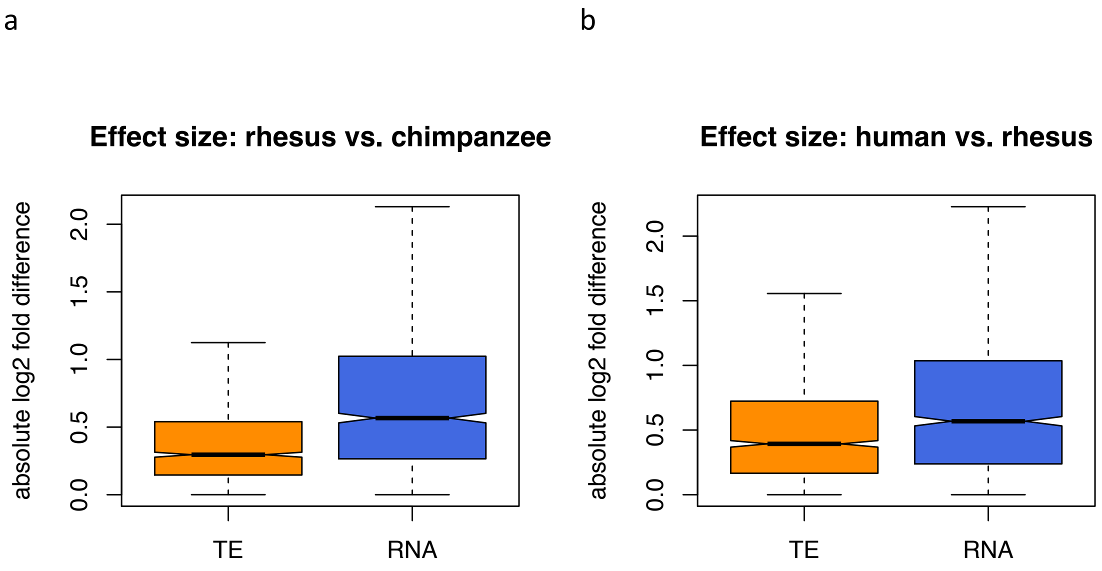

Figure 2 S3 Inter-species divergence at the transcript level is greater than that of translation efficiency. Boxplots comparing effect size (absolute $\log_2$ ratio) of inter-species divergence (TE: translation efficiency, RNA: transcript level). Only genes that are diverged at the protein level were included in this analysis. (a) rhesus macaque vs. chimpanzee (b) rhesus macaque vs. human.

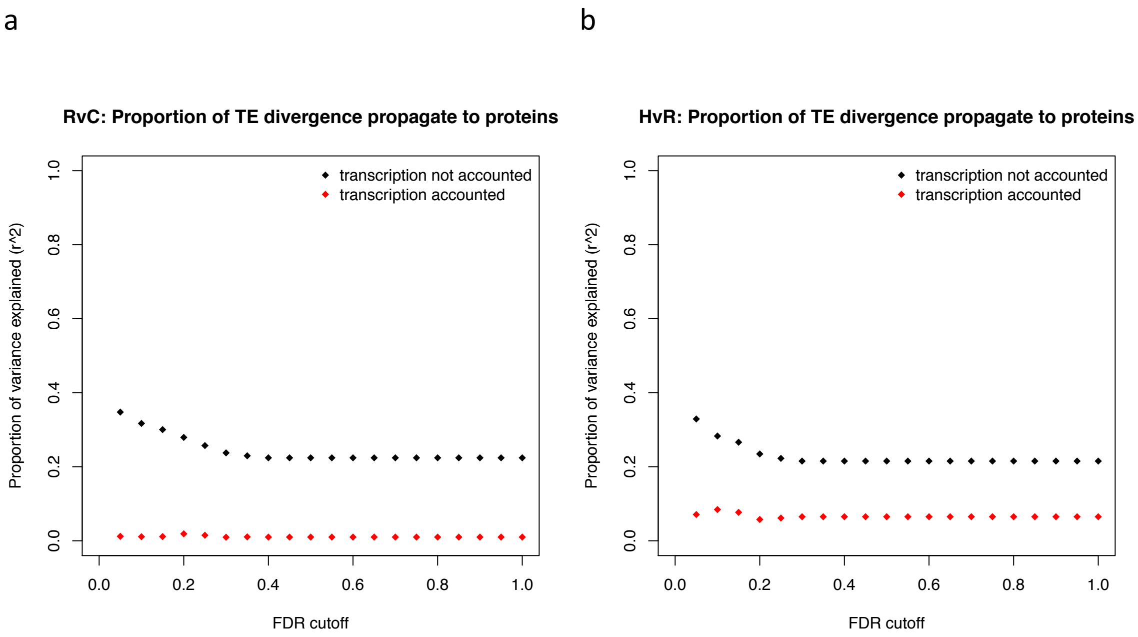

Figure 2 S4 Inter-species divergence in translation efficiency rarely contributes to inter-species divergence in protein level. Proportion of inter-species divergence propagated from translation level to the protein level (y-axis) was estimated using coefficient of determination (r^2) between translation level divergence and protein level divergence. Divergence between species at each level for each gene was estimated using the regression coefficient of the species term of each respective linear model (see Methods). Each r^2 was calculated for a subset of genes that were defined by an FDR cutoff (x-axis) for divergence in protein level. These coefficients (r^2) were calculated either before (black) or after (red) the effects from the transcript level were regressed out (a) rhesus macaque vs. chimpanzee (b) rhesus macaque vs. human.

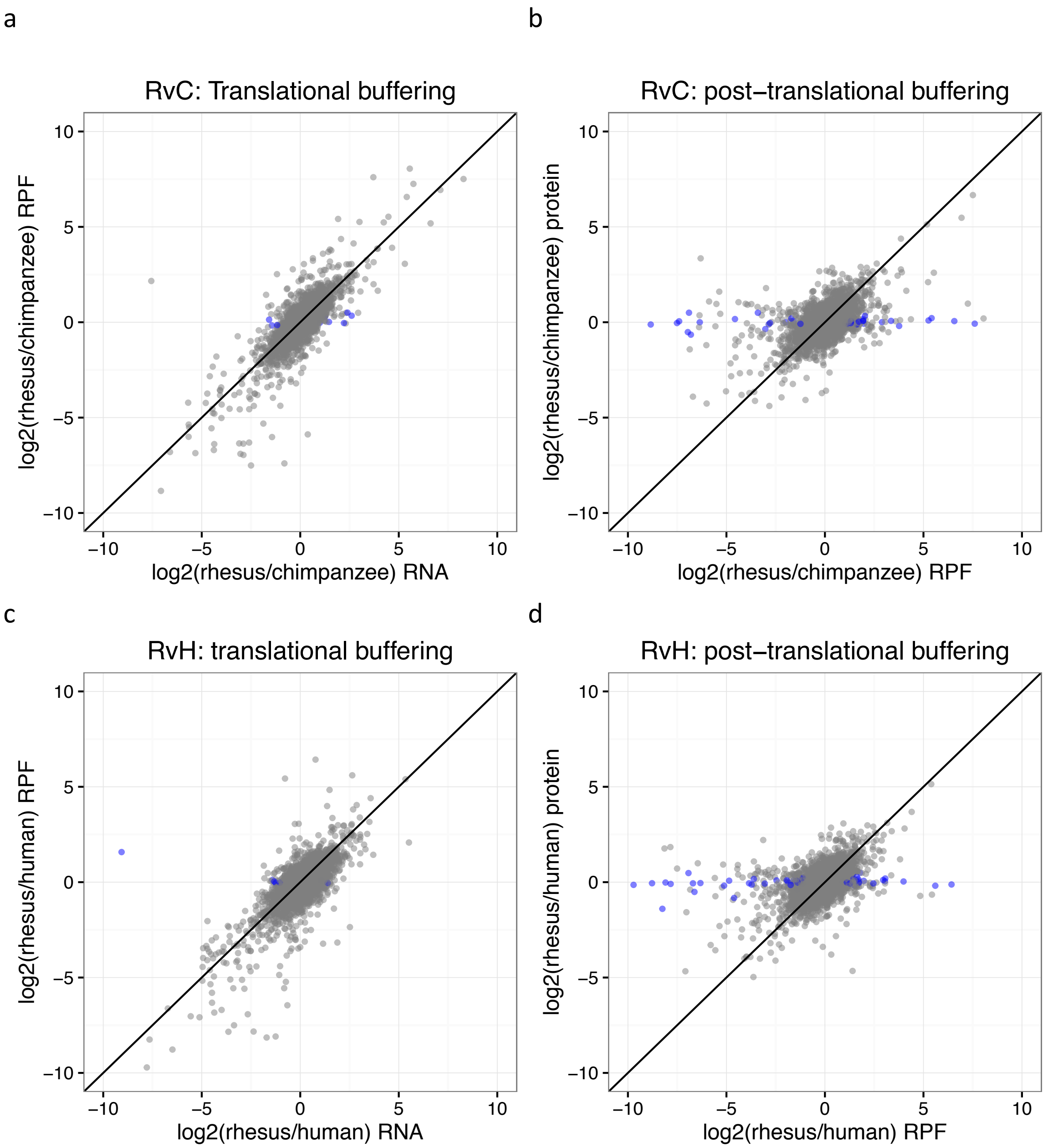

Figure 3 S1 Scatter plots of inter-species divergence comparing between different molecular traits (RNA: transcript level, RPF: level of translation, protein: protein level). Each data point represents a gene, and the position along each axis indicates log_2 ratio of the two species in comparison for each molecular trait. The color of each data point indicates whether the inter-species divergence for each gene is significantly buffered at the downstream molecular trait at

a significance cutoff of FWER 0.05 (blue: significant, grey: not significant). (a, b) RvC: rhesus macaque vs. chimpanzee. (c, d) RvH: rhesus macaque vs. human.

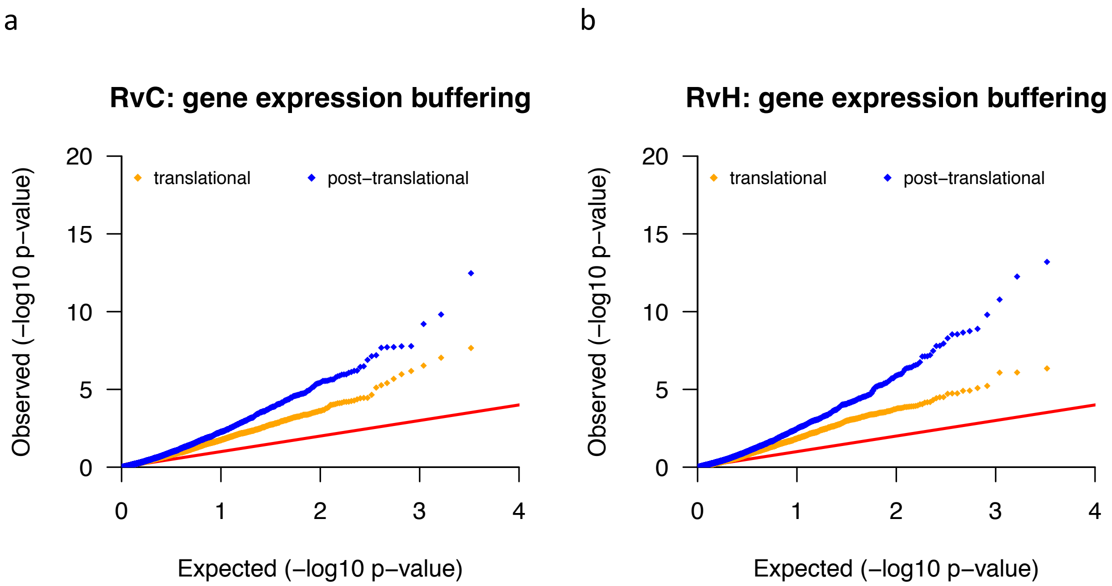

Figure 3 S2 Post-translational buffering of inter-species divergence in gene expression occurs much more frequently than translational buffering. Quantile-quantile plots of $-\log_{10}$(p-values) derived from testing for buffering of inter-species divergence (orange: translational buffering, blue: post-translational buffering). Observed p-values (y-axis) were plotted against the null expectation (i.e. uniform distribution of p-values) (x-axis). The red line marked the expected results from a scenario where no buffering was observed. (a) RvC: rhesus macaque vs. chimpanzee, (b) RvH: rhesus macaque vs. human.

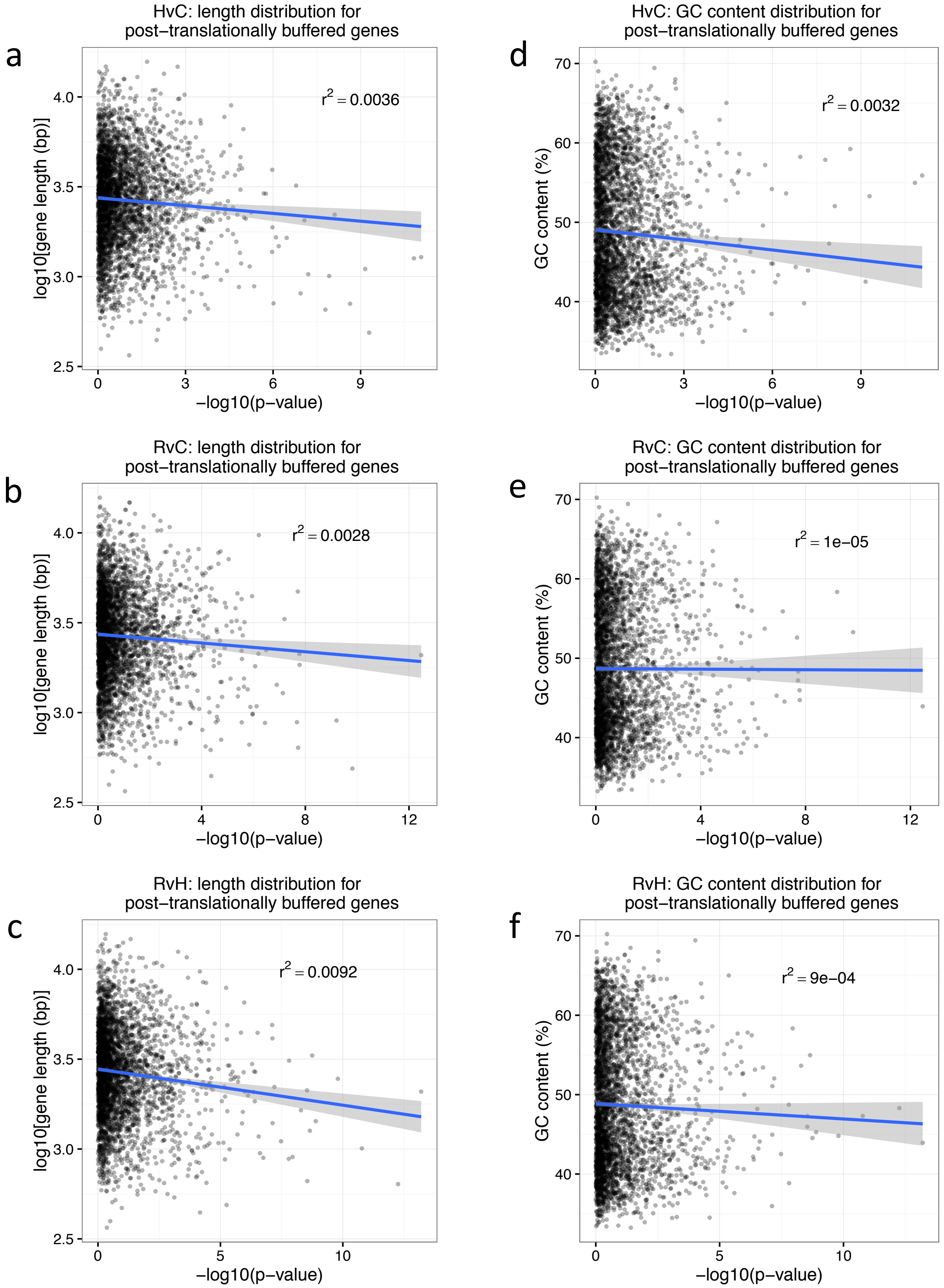

Figure 3 S3 Post-translationally buffered genes are not enriched for longer genes, nor are they enriched for genes with higher GC content. In each scatter plot, $-\log_{10}$(p-value) derived from

testing for post-translational buffering of inter-species divergence in gene expression were plotted against either gene length (a, b, c) or GC content (d, e, f). Each data point represented a gene and all genes quantified in this study were included. HvC: human vs. chimpanzee, RvC: rhesus macaque vs. chimpanzee, RvH: rhesus macaque vs. human.

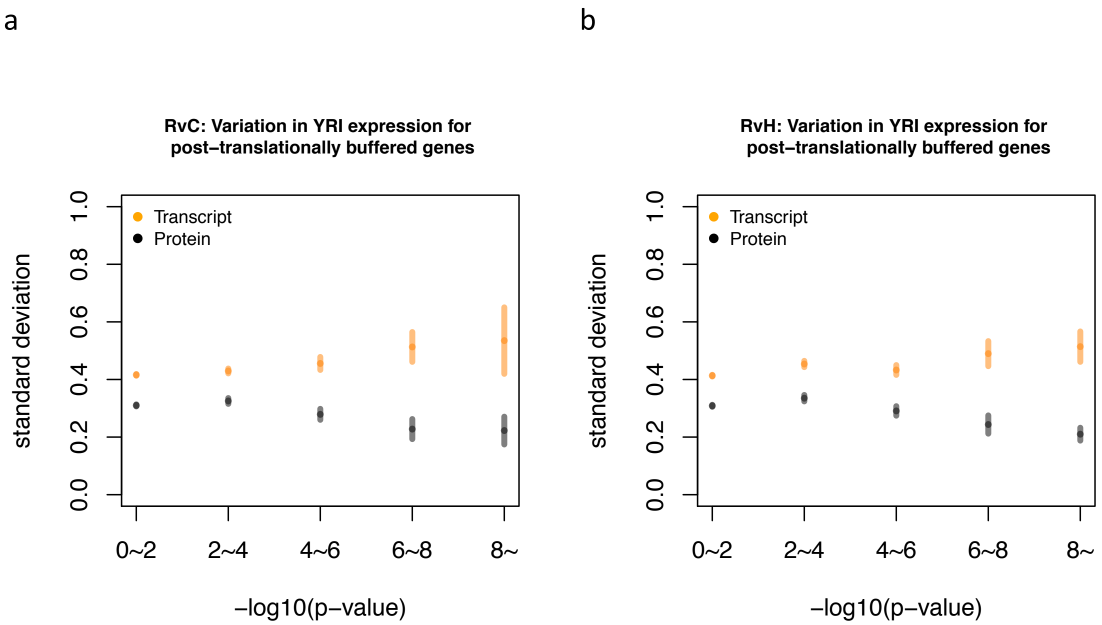

Figure 3 S4 Potential impacts of post-translational buffering on relaxation of transcriptional regulation. Buffering of inter-species divergence is more likely to occur to genes that have a higher within-species (human) transcript level variation. Standard deviation across YRI individuals of transcript level (orange) or that of protein level (black) was plotted against significance level of inter-species post-translational buffering. Individual genes were grouped into bins according to their significance level of inter-species post-translational buffering (x-axis). Position along the y-axis for each bin indicated mean ± standard error. (a) RvC: rhesus macaque vs. chimpanzee, (b) RvH: rhesus macaque vs. human.

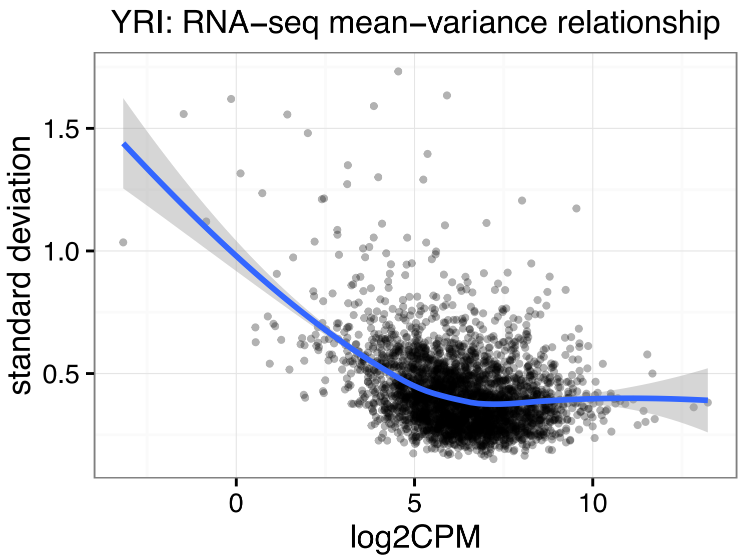

Figure 3 S5 A scatter plot showing the mean-variance relationship in YRI RNA-seq data. Each data point represents a gene. Position along the x-axis indicated the expression level (averaged across individuals) and position along the y-axis indicated the standard deviation. The blue trend line and the corresponding shaded area (95% confidence interval) are estimated using a loess fit.

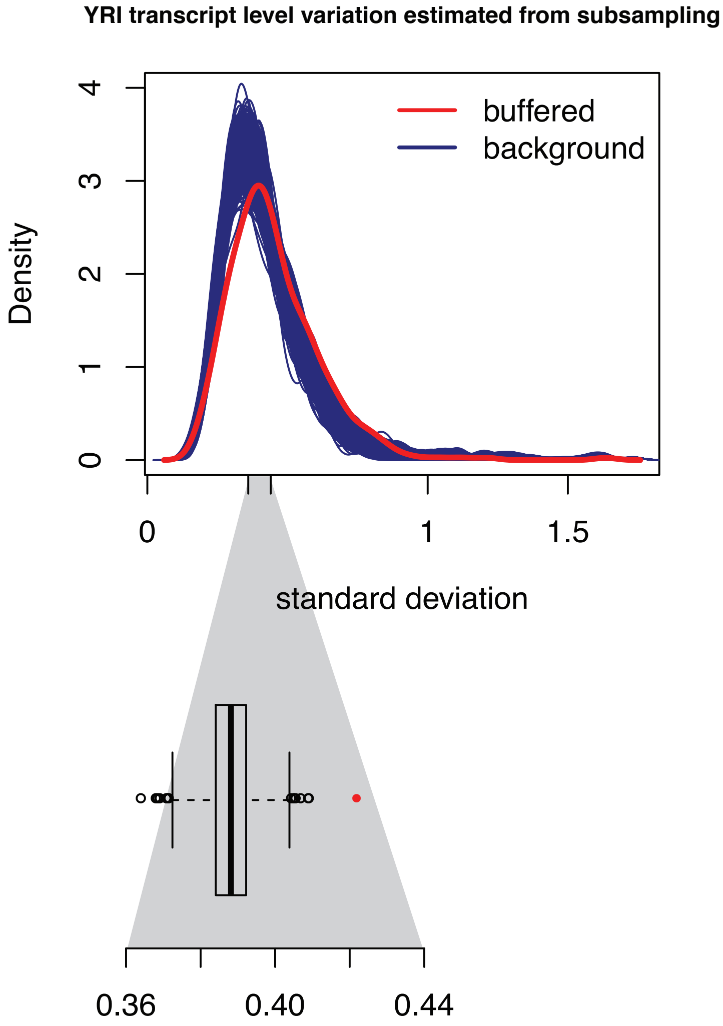

Figure 3 S6 Further subsampling analysis confirmed the observed higher transcript level variation in YRI population for human-chimpanzee post-translationally buffered genes. To account for expression level differences between buffered genes and background genes, subsets of background genes, each matched for the transcript expression level distribution of the buffered genes, were sampled from the full set of background genes. Top: A density plot showing distributions of standard deviations of transcript level of YRI individuals (red: buffered genes, blue: subsamples from the background genes). Bottom: A boxplot summarizing the median value of standard deviations of each subsampled group of genes presented in the top

plot. The red data point indicated the median value of standard deviations of post-translationally buffered genes.
